# Multiomics Data Analysis and Identification of Immune-Related Prognostic Signatures With Potential Implications in Prognosis and Immune Checkpoint Blockade Therapy of Glioblastoma

**DOI:** 10.3389/fneur.2022.886913

**Published:** 2022-05-20

**Authors:** Shuai Ma, Fang Wang, Nan Wang, Jiaqi Jin, Yixu Ba, Hang Ji, Jianyang Du, Shaoshan Hu

**Affiliations:** ^1^Department of Neurosurgery, The Second Affiliated Hospital of Zhengzhou University, Zhengzhou, China; ^2^Department of Neurosurgery, Emergency Medicine Center, Zhejiang Provincial People's Hospital Affiliated to Hangzhou Medical College, Hangzhou, China; ^3^Department of Neurosurgery, The Second Affiliated Hospital of Harbin Medical University, Harbin, China; ^4^Department of Neurosurgery, Shandong Provincial Hospital Affiliated to Shandong First Medical University, Jinan, China

**Keywords:** glioblastoma, immune gene, mutation, methylation, PD-1

## Abstract

**Background:**

In recent years, glioblastoma multiforme (GBM) has been a concern of many researchers, as it is one of the main drivers of cancer-related deaths worldwide. GBM in general usually does not responding well to immunotherapy due to its unique microenvironment.

**Methods:**

To uncover any further informative immune-related prognostic signatures, we explored the immune-related distinction in the genetic or epigenetic features of the three types (expression profile, somatic mutation, and DNA methylation). Twenty eight immune-related hub genes were identified by Weighted Gene Co-Expression Network Analysis (WGCNA). The findings showed that three genes (*IL1R1, TNFSF12*, and *VDR*) were identified to construct an immune-related prognostic model (IRPM) by lasso regression. Then, we used three hub genes to construct an IRPM for GBM and clarify the immunity, mutation, and methylation characteristics.

**Results:**

Survival analysis of patients undergoing anti-program cell death protein 1 (anti-PD-1) therapy showed that overall survival was superior in the low-risk group than in the high-risk group. The high-risk group had an association with epithelial-mesenchymal transition (EMT), high immune cell infiltration, immune activation, a low mutation number, and high methylation, while the low-risk group was adverse status.

**Conclusions:**

In conclusion, IRPM is a promising tool to distinguish the prognosis of patients and molecular and immune characteristics in GBM, and the IRPM risk score can be used to predict patient sensitivity to checkpoint inhibitor blockade therapy. Thus, three immune-related signatures will guide us in improving treatment strategies and developing objective diagnostic tools.

## Background

Despite the tremendous improvements in the comprehensive treatment of glioblastoma (GBM), the 5- and 10-year survival rates for GBM still remain at 5 and 2.6%, respectively (1). Furthermore, treatment strategies for GBM are limited and there is an urgent need to develop novel tools to forecast patient survival (2, 3).

In recent years, different approaches for GBM have been investigated, among which immunotherapy is one of the most attractive approaches and is currently undergoing active research. Patients treated with immune checkpoint blockade therapy (ICB) have shown a longer survival than those receiving conventional therapy (4, 5). But, the treatments are effective in only a minority of patients, most patients have a limited or no response to treatment, especially during GBM progression. There is an immediate need to comprehensively understand the tumor microenvironment of GBM and identify a worthy prognostic model for predicting the benefit of immunotherapy for GBM patients.

In our study, we merged 2,138 immune-related genes from the ImmPort and the InnateDB databases with all transcriptomes of GBM from The Cancer Genome Atlas (TCGA) database to obtain 1,439 genes. which were used for Weighted Gene Co-Expression Network Analysis (WGCNA), univariate regression, and lasso regression to construct the immune-related prognostic model (IRPM) (6, 7). We further validated the IRPM on the Chinese Glioma Genome Atlas (CGGA) database, IRPM was found to be a good predictor of patient survival time in two cohorts. Then, we performed anti-program cell death protein 1 (anti-PD-1) validation on IRPM in the GSE78220 cohort and obtained consistent results for survival. Finally, we checked the immune infiltration, mutation, and methylation status of IRPM which showed that IRPM could also respond to the tumor microenvironment of GBM (8).

## Methods

### Data Collection and Disposal

The RNA_seq profiles (FPKM) of 169 GBM samples and their clinicopathological data (Supplementary Table 1) were gathered from the TCGA database (https://portal.gdc.cancer.gov/). RNA_seq of normal brain samples were downloaded from the GTEx database (https://gtexportal.org/home/). FPKM values were then converted to TPM values. The “ComBat” algorithm using the sva package corrects for batch effects from non-biotechnology bias (9). A total of 35,654 differential expressed genes (DEGs) were obtained from 169 tumors and 100 normal samples. All patients without prognostic information were initially excluded. The content of immune-related genes (2,138) was gathered from the ImmPort and InnateDB databases. A higher tumor immune dysfunction and exclusion (TIDE) score (Supplementary Table S2) was calculated online (https://tide.dfci.harvard.edu/) and an 18-gene T-cell inflammatory marker (TIS) score was calculated as an average value of log2-scale normalized TPM expression of the 18 signature genes (10). PD-1 validation information was obtained from the GSE78220. The ESTIMATE algorithm using R scripts version 3.6.3 loaded with the estimate package was applied to estimate the ratio of the immune-stromal component in TME for each sample (11). The calculated results were presented in three types of scores: Immunescore, Stromalscore, and Estimatescore (Supplementary Table S3). The prognostic capability of the IRPM was validated in the CGGA database.

### WGCNA

Weighted Gene Co-Expression Network Analysis was applied to recognize the pivotal genes among 1,439 immune-related genes. First, to conform our gene distribution to the scale-free network, we constructed the adjacency matrix based on the connectivity of the optimal β value and transformed the adjacency matrix into a topology overlap matrix (TOM). Next, the heterogeneity among genes was applied to aggregate the genes for the TOM we have acquired. Eventually, the identified TOMs are defined as components, and stratified clustering is performed using a dynamic tree-cutting algorithm to identify modules with a minimum module size of 25 (12, 13). To further investigate the correlation between clinical criteria and module Eigengenes (MEs) in each module, the *p*-values were defined as module eigengenes (MMs) by the hypergeometric test of the overlap of the design parameters with the merged modules. Gene significance (GS) was considered as the relevance of these parameters to the expression pattern of MEs. The central genes of the most associated modules were identified under the MM >0.6 and GS >0.5 thresholds. The clusterProfiler package was used to study the biological processes of phenotype-related genes (14). Copy number variation (CNV) data were visualized using the “RCircos” package. Genomic Identification of Important Targets in Cancer algorithm was used to classify CNV status from gains to losses. The parameter thresholds for both genomic gain and loss were set to 0.2 and −0.2. Twenty eight hub gene CNV data were compared in high and low-risk groups using a chi-square test (15).

### Immunohistochemistry

Immunohistochemistry staining has proceeded as described previously (16). Histochemical scores were computed using the Quant Center analysis tool:


$$\begin{array}{l} Histochemistry\;score\;=\;\overset{n}{\underset{i\;=\;1}{\sum }}PI\;*i \end{array}$$

in which PI is the proportion of cells of various intensities and the corresponding coefficients (i) (17).

### Construction and Identification of the Immune-Related Signature

To recognize independent prognostic genes, we carried out a univariate Cox regression analysis of 28 hub genes (Supplementary Table S4). Genes with *p*-values < 0.05 were selected and the potential prediction model was constructed by a lasso regression algorithm. Subsampling was performed using 1,000 cross-validation in a 7:3 ratio from the training set. Finally, three genes and their regression coefficients were acquired. The riskscore was used in the formulae:


(1) $$\begin{array}{r} \text{Riskscore}=\overset{n}{\underset{i=\;1}{\sum }}\text{Coef}*exp \end{array}$$

in which Coef was the coefficient and exp was the expression value (18). In the following, we used risk instead of risk score. To explore whether IRPM could independently predict OS, immunotherapy, mutation, and methylation. We separated the groups into high and low score groups according to median risk scores. The K-M and log-rank tests were applied to compare OS between the two groups. The percentage of gender, GBM molecular subtype, *IDH1* status, age, and sample type between the high and low-risk groups were examined by chi-square tests. The area under the curve (AUC) was determined to evaluate the predictive reliability of model (19).

### Immune Infiltration

The relative infiltration of 28 immune cells in TME was characterized by applying ssGSEA. It was obtained from a recent paper for a set of characteristic genes for each immune cell type (20). In ssGSEA analysis, each immune cell type showed an enriched fraction in terms of relative abundance.

### Analyses of Mutations Among Subgroups

To reveal related genetic alterations, including single nucleotide variants (SNV), single nucleotide polymorphisms (SNP), insertions (INS), and deletions (DEL), MuTect2 was used to identify default parameters based on a two-by-two comparison file (tumor and matched germline). Mutation datasets were analyzed and visualized using the “maftools” R package for both groups (21).

### Methylation Analyses

For DNA methylation, Illumina Infinium 450 k DNA methylation array data were processed using the R package “ChAMP.” Samples with over 20% missing values were excluded, and a total of 169 samples were taken and subdivided into two groups according to the above riskscore groups. The remaining missing values were counted with the imputation function of ChAMP. The β values were standardized using a peak-based correction. Additionally, the differentially methylated probes (DMPs) and regions were determined separately using the limma package (8).

### Statistical Analyses

Glioblastoma multiforme expression levels were compared in terms of age, sex, healthy samples, primary and recurrent GBM, *IDH1* status, and cytosine-phosphoguanine island methylation phenotype (G-CIMP) status using the chi-square test. The distribution of GBM subtypes (classic, mesenchymal, neural, and proneural) was compared using one-way ANOVA. To assess the forecast reliability of the prognostic model, we drew ROC curves and calculated the AUC.

## Results

### Schematic Diagram of the Study Design

**Figure 1:**
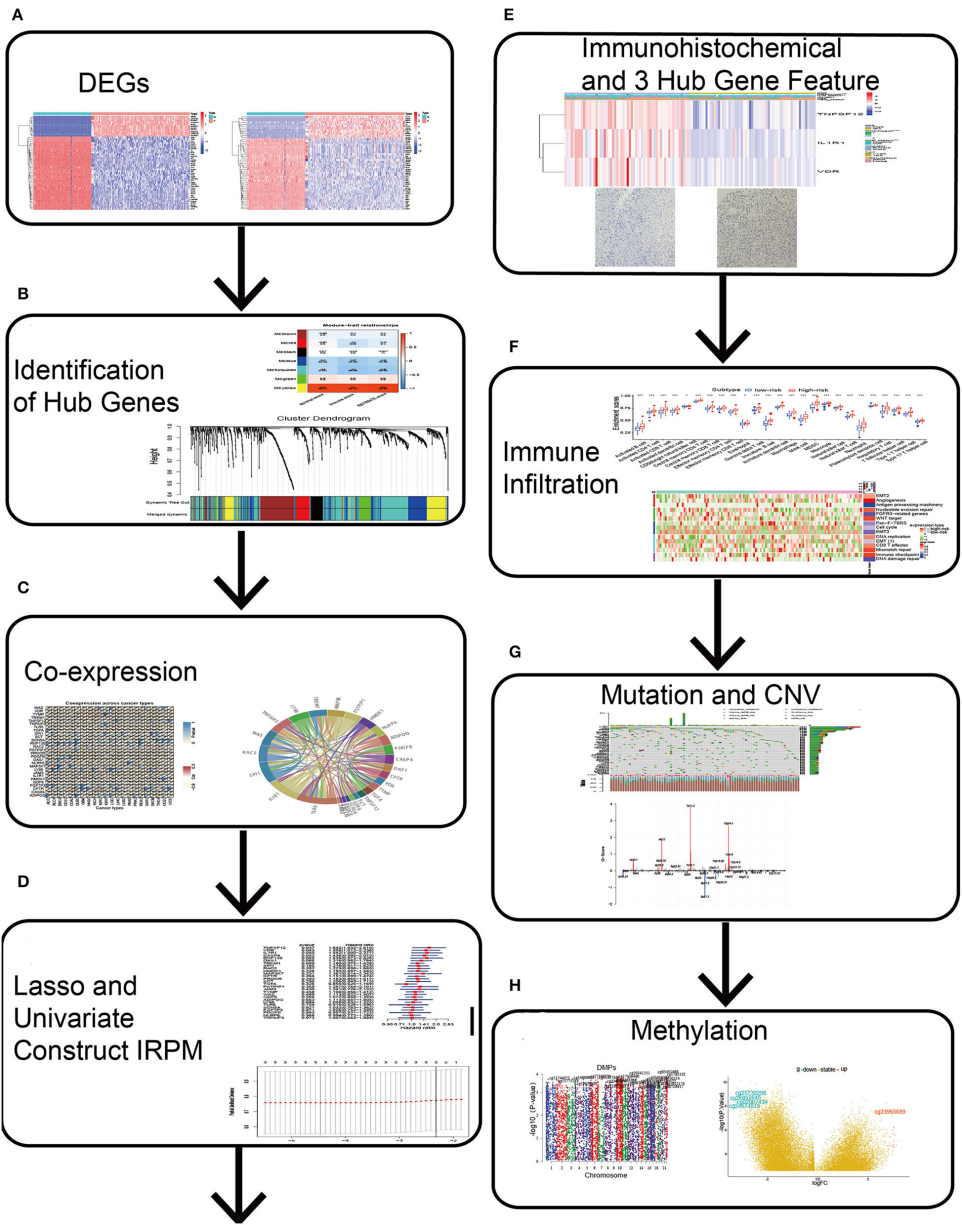
Schematic diagram of this study. **(A)** Differential genes were obtained from GBM and normal brain tissue. **(B)** Hub genes were identified by Weighted Gene Co-Expression Network Analysis (WGCNA). **(C)** Clinicopathologic characteristics of 28 hub genes were analyzed. **(D)** Lasso and univariate regression were used to identify 3 key genes and the corresponding risk coefficients. **(E)** ICH and TIMER were used to analyse the clinicopathological characteristics of three key genes. **(F)** ssGSEA was used to analyse immune infiltration in both groups. **(G)** Maftools and Genomic Identification of Significant Targets in Cancer algorithm were used to analyse mutations and CNV in both groups. **(H)** ChAMP was used to analyse methylation in high and low-risk groups.

Differentially expressed genes between normal and GBM samples were merged with immune-related genes (Figure 1A). Simultaneous “WGCNA,” univariate, and lasso regression were performed to obtain three immune-related signatures (Figures 1B–D), which were validated by immunohistochemistry (Figure 1E). Finally, we further constructed IRPM and analyzed the multi-omic data of immune infiltration, mutation, and methylation in IRPM (Figures 1F–H).

### Immune-Related Hub Genes

By intersecting these DEGs (35,654 genes) with the immune-related genes (2,138 genes), we obtained 1,439 immune-related genes and presented a heatmap for the two sets of DEGs (35,654 and 1,439 genes) between GBM and normal samples separately (Supplementary Figures S1A,B), as well as supplied GO and KEGG enrichment analysis for 35,654 and 1,439 DEGs (Supplementary Figures S1C–F). The results showed that the top 10 GO terms and KEGG pathways of 1,439 genes were mainly focused on immune-related pathways, further revealing the importance of these immune-related genes.

**Figure 2:**
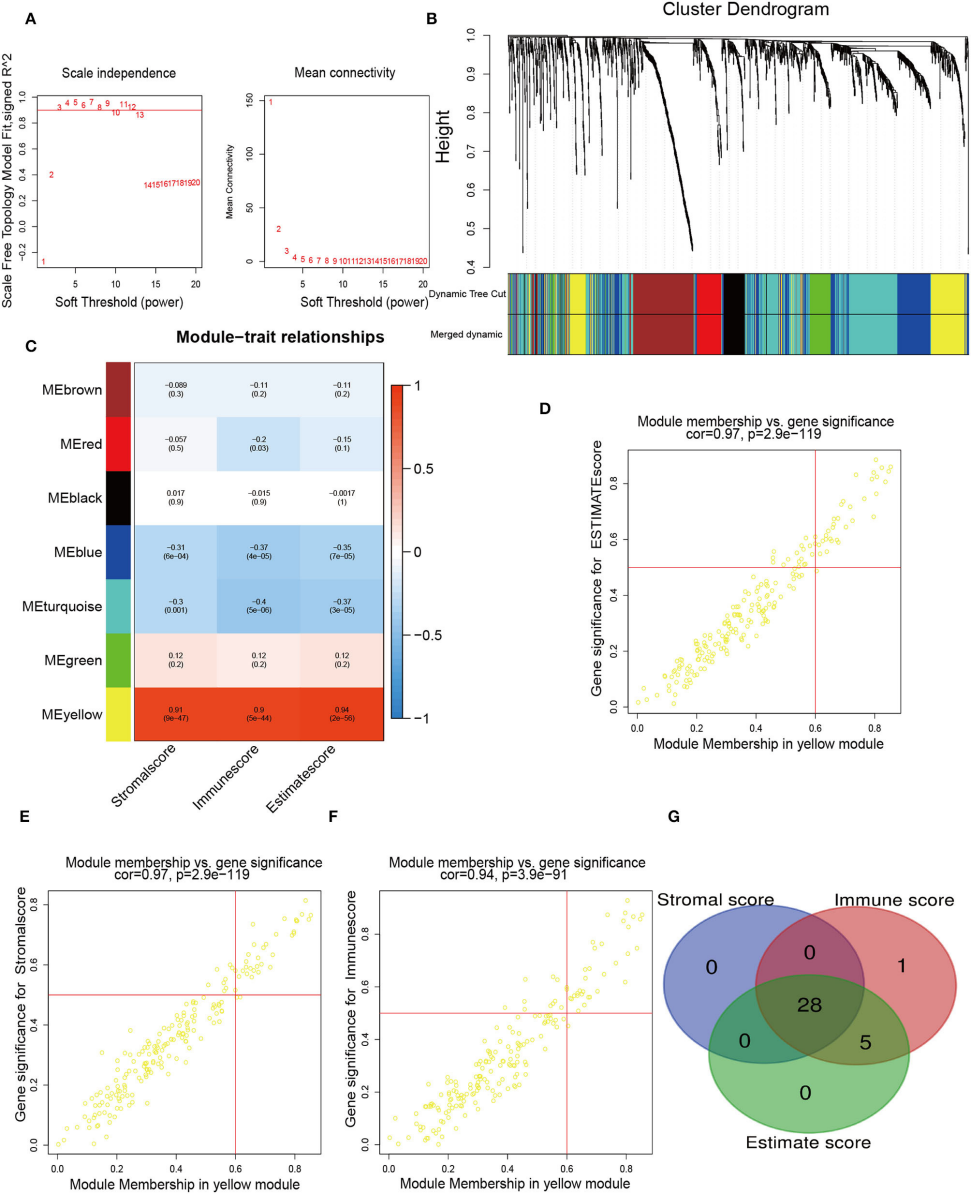
Identification of immune-related hub genes. **(A)** WGCNA process of immune-related differentially expressed genes with a soft threshold β = 8. **(B)** Hierarchical clustering dendrogram of the co-expressed genes identified in the modules. Cluster dendrograms have branches corresponding to different gene modules. A clustered dendrogram has one gene corresponding to each leaf. **(C)** Relevance of gene modules to clinical traits. For each cell, the correlation coefficient corresponds to the relevance between the gene module and the clinical trait, which changes from red to blue. The homologous *p*-values are also annotated. **(D**–**F)** The eigengenes in the yellow module are shown in separate alternate scatter plots. **(G)** A Venn diagram shows the overlapping genes among genes grouped according to Stromalscore, genes grouped based on immunescore, and genes grouped according to Estimatescore.

To determine the pivotal nodes of these genes, which were introduced into the WGCNA. To confirm our gene assignments to the scale-free network, we established the adjacency matrix according to the connectivity based on an optimal β value. The optimal beta value is found when the level of scale independence is set to 0.8 (Figure 2A). The gene dendrogram was generated by mean linkage hierarchical clustering. The colored rows on the bottom of the tree diagram showed the module assignments determined by dynamic tree cutting (Figure 2B). On this basis, the heatmap plot the adjacencies in the eigengene network with the trait weight. The heatmap had one modular trait gene (marked in color) or weight for every row and column. Immune-related genes were distributed to 21 modules. Notably, we found that the yellow module possessed a significant correlation with Stromalscore, Immunescore, and Estimatescore, with *p*-values of 0.87, 0.95, and 0.95, respectively (Figure 2C). Finally, we selected the genes of this module for sequential analysis, and the pivotal genes with MM >0.6 and GS >0.5 were screened (Figures 2D–F). To further identify the immune-related signatures, we applied the Venn Diagram web tools and acquired 28 hub genes among genes grouped according to Stromalscore, Immunescore, and Estimatescore (Figure 2G). The above results implied that the intersection of these genes in three groups is important for our further understanding of immune genes. These identified genes may lead us to pave the way for more precise analysis.

### Clinicopathologic Characteristics of 28 Hub Genes

**Figure 3:**
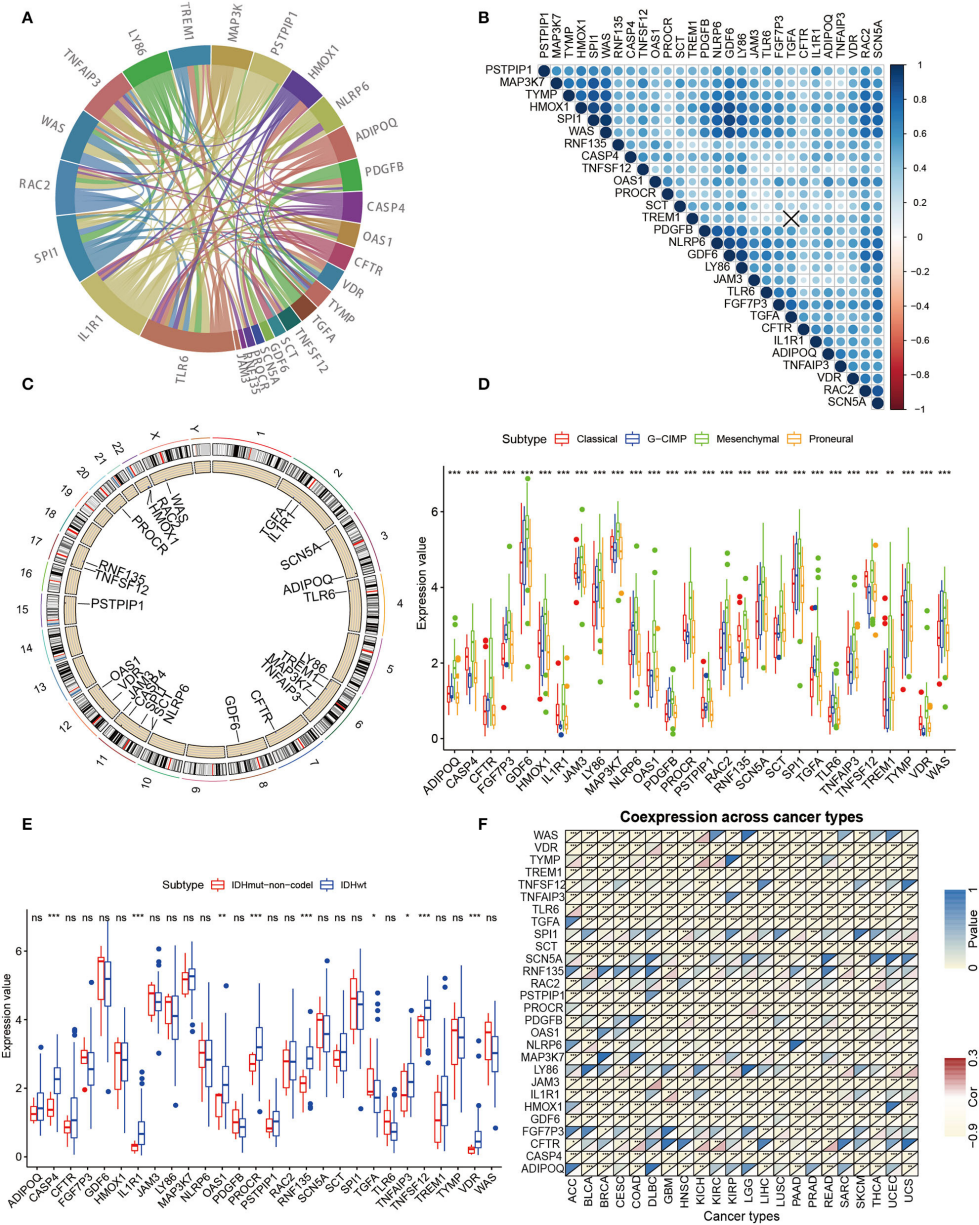
Further analysis of clinicopathologic characteristics of 28 hub genes. **(A)** PPI analysis was performed among the 28 hub genes. **(B)** Spearman relevance analysis of the 28 hub genes. **(C)** Circle maps of disparity CNV of pivotal genes. The black dots outside the circle represent amplification, while the red dots inside the circle represent deletion. **(D)** Expression of 28 hub genes in GBM molecular typing. **(E)** Differential expression of hub genes in GBM IDH typing is evident for all 28 genes. **(F)** Correlation of the expression value of hub genes and Immunescore for pan-cancer (Pearson test).

To further investigate the interaction pattern characteristics of these 28 hub genes, a PPI network analysis was performed (Figure 3A). For the relevance networks of hub genes, *SCN5A* maintained a distinct correlation with *GDF6*, and *LY86* also had a distinct correlation with *WAS, SPI1*, and *HMOX1* (*p* < 0.05, Figure 3B). We ascertained the alterations of the 28 immune-related genes featuring CNV on the chromosome. These results indicated that the CNV status of these 25 genes is relevant to the progression and occurrence of glioma (Figure 3C). Next, the correlation of hub gene expression patterns with clinicopathology was investigated in our study. All hub genes showed significant differences in the four molecular subgroups (classical, neural, mesenchymal, proneural, Figure 3D) (22–25). Furthermore, significant differences in *CASP4, IL1R1, OAS1, PROCR, RNF135, TGFA, TNFAIP3, TNFSF12*, and *VDR* were observed in the *IDH* mutant molecular subtype grouping (Figure 3E). Finally, a pan-cancer analysis of hub genes was performed and the hub genes showed a significant negative correlation in 22 tumors (Figure 3F). Through multi-omics data, we found that these hub genes may play a crucial role in the development of GBM.

### Univariate Regression and Lasso Regression to Identify Immune-Related Prognostic Genes and Construct Risk Models

To identify immune-related prognostic genes, we executed univariate regression on the 28 hub genes and found that three genes (*IL1R1, TNFSF12*, and *VDR*) were statistically correlated with overall survival (OS; Supplementary Figure S2A). Next, we performed lasso regression on these three genes to obtain the regression coefficients of the three genes (Supplementary Figures S2B,C) and constructed the IRPM. We observed a significant difference in survival between the two groups of GBM patients (Supplementary Figure S2D). The same result was also observed in the classical GBM molecular typing (Supplementary Figure S2E). To verify the precision of the IRPM in predicting the prognosis, the ROC was calculated for the AUC of 3 and 5-year in the TCGA cohort, and the results revealed that the AUCs were .799 (3-year) and 0.771 (5-year) and were the highest among other clinical characteristics (Supplementary Figures S2F,G). We analyzed the proportion of IDH1 mutations in the two groups and found that all the high-risk groups were WT types, while the low-risk groups showed a certain proportion of Mutation types (Supplementary Figure S2H). This may also be a reason for the long survival time of patients in the low-risk group. Finally, the IRPM was validated using the CGGA database. The results also showed a significant distinction between the two groups (*p* < 0.05) (Supplementary Figure S2I). The AUC of the riskscore was 0.709 (3-year) and 0.749 (5-year) in the CGGAmRNA_325 dataset. In the CGGAmRNA_693 dataset, the AUCs were 0.709 (3-year) and 0.728 (5-year) (Supplementary Figures S2J–M). We analyzed the proportion of IDH1 mutations and recurrence status in the two groups and found that the proportion of mutation types was higher in the low-risk group than in the high-risk group, and there was little distinction in the recurrence status between the high and low-risk groups in CGGA_325 cohort (Supplementary Figures S3A,B). The proportion of mutation type and recurrence status was higher in the low-risk group than in the high-risk group in the CGGA_693 cohort (Supplementary Figures S3C,D). The above findings showed that the IRPM had excellent accuracy and could accurately forecast the survival time of patients.

### Clinical Characteristics of *TNFSF12, VDR*, and *IL1R1*

**Figure 4:**
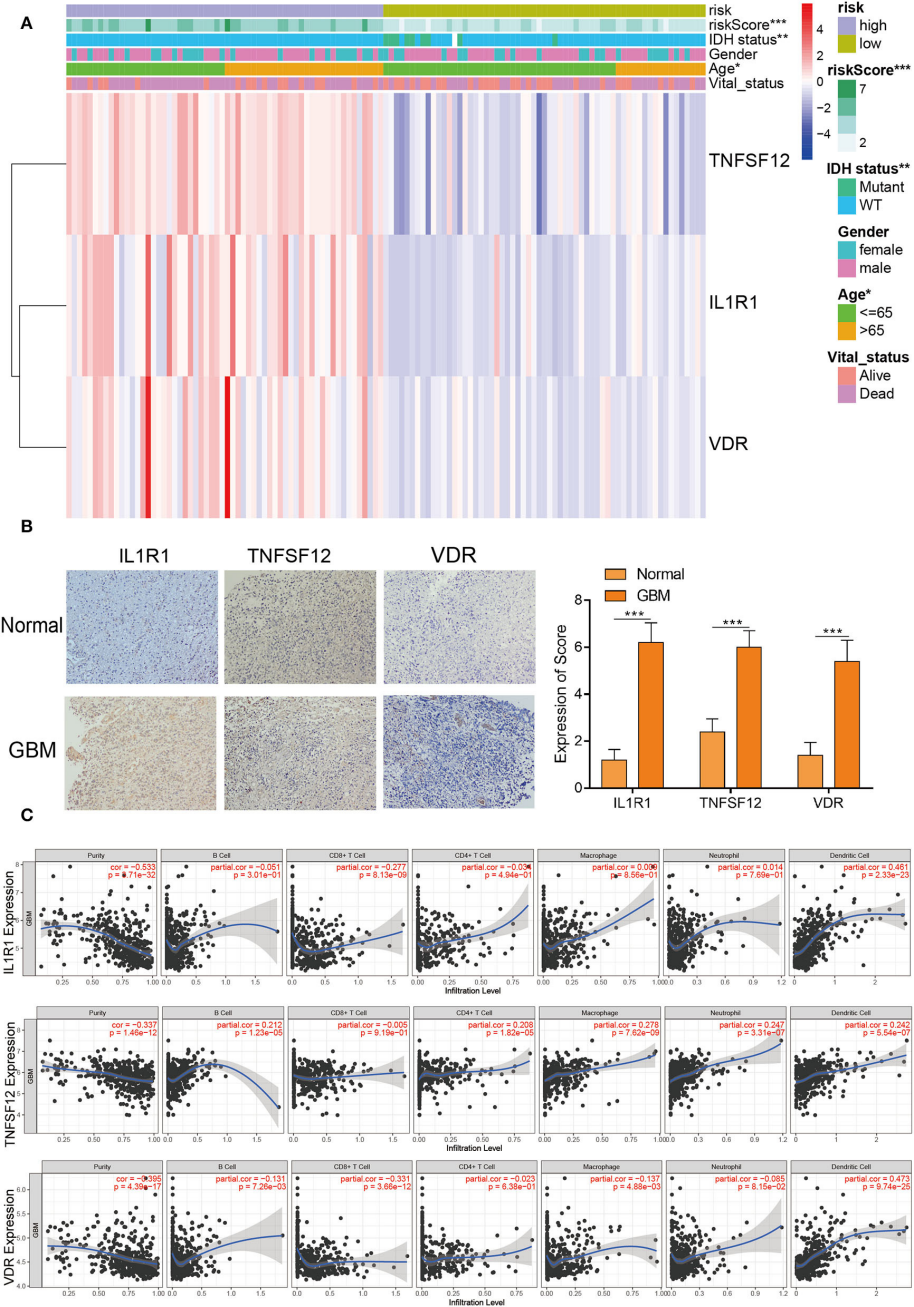
Clinicopathological characteristics and immune infiltration of three hub genes. **(A)** Heatmap risk clusters with clinical and molecular pathological parameters. **(B)** The protein expression of *VDR, TNFSF12*, and *IL1R1* in GBM and normal tissues were tested by IHC. Whole tissue photos are shown (100 × and 400 × ). **(C)** The relevance between the expression levels of *VDR, TNFSF12*, and *IL1R1* and the infiltration of macrophages, dendritic cells, and B cells in GBM.

The heatmap with clinicopathological characteristics of IRPM was analyzed. The results showed that there were statistically significant distinctions in riskscore, *IDH1* status, and age between both groups (*p* < 0.05) (Figure 4A). Moreover, pan-cancer analysis using the GEPIA website for *TNFSF12, VDR*, and *IL1R1* revealed that these three genes had significant expression differences between multiple tumors and normal tissues, and the combined results revealed that *TNFSF12, VDR*, and *IL1R1* also had significant prognostic diversity for multiple tumors (Supplementary Figures S4A–C). Further validation of protein expression of these three pivotal genes, we performed immunohistochemical experiments on the *IL1R1, TNFSF12*, and *VDR* genes in human specimens. There were significantly higher expression levels of VDR, TNFSF12, and IL1R1 proteins in tumor samples than in normal samples (Figure 4B), consistent with the results of the above bioinformatics analyses. Thus, these proteins may have an essential influence on the prognosis of tumors. Furthermore, IL1R1 had a significant positive correlation with dendritic cells and a significant negative correlation with CD8+ T cells, TNFSF12 had a significant positive correlation with macrophages, dendritic cells, and neutrophils, and VDR had a significant positive correlation with dendritic cells and CD8+ T cells (Figure 4C). These findings revealed that these three genes were crucial for tumor immunity. Significant biomarkers that reflect the response to checkpoint blockade immunotherapy could be broadly classified into two types: biomarkers associated with the burden of neoepitopes, such as MSI or TMB, and inflammatory infiltrating TIME. The radar plots revealed that 18 of 33 tumors had a remarkable relevance between *IL1R1* and TMB and 4 of 33 tumors had a significant relevance between the *TNFSF12* and TMB, and 11 of 33 tumors were significantly associated with *VDR* and TMB (Supplementary Figures S5A–C). Next, we analyzed the correlation between MSI and these three genes (Supplementary Figures S5D–F). We found that HNSC and STAD displayed the largest negative relevance with both *IL1R1* and MSI, LUSC, STAD, and THCA revealed the largest negative relevance with both *TNFSF12* and MSI, and HNSC exhibited the largest negative relevance with both *VDR* and MSI. These results could potentially reflect that the number of lymphocytes in these tumors was too low or excessive. The above results revealed that high expression of the three genes might be a driver of GBM and play an essential role in GBM immunity.

### Immune Landscape Related to Histopathologic Characteristics of IRPM

**Figure 5:**
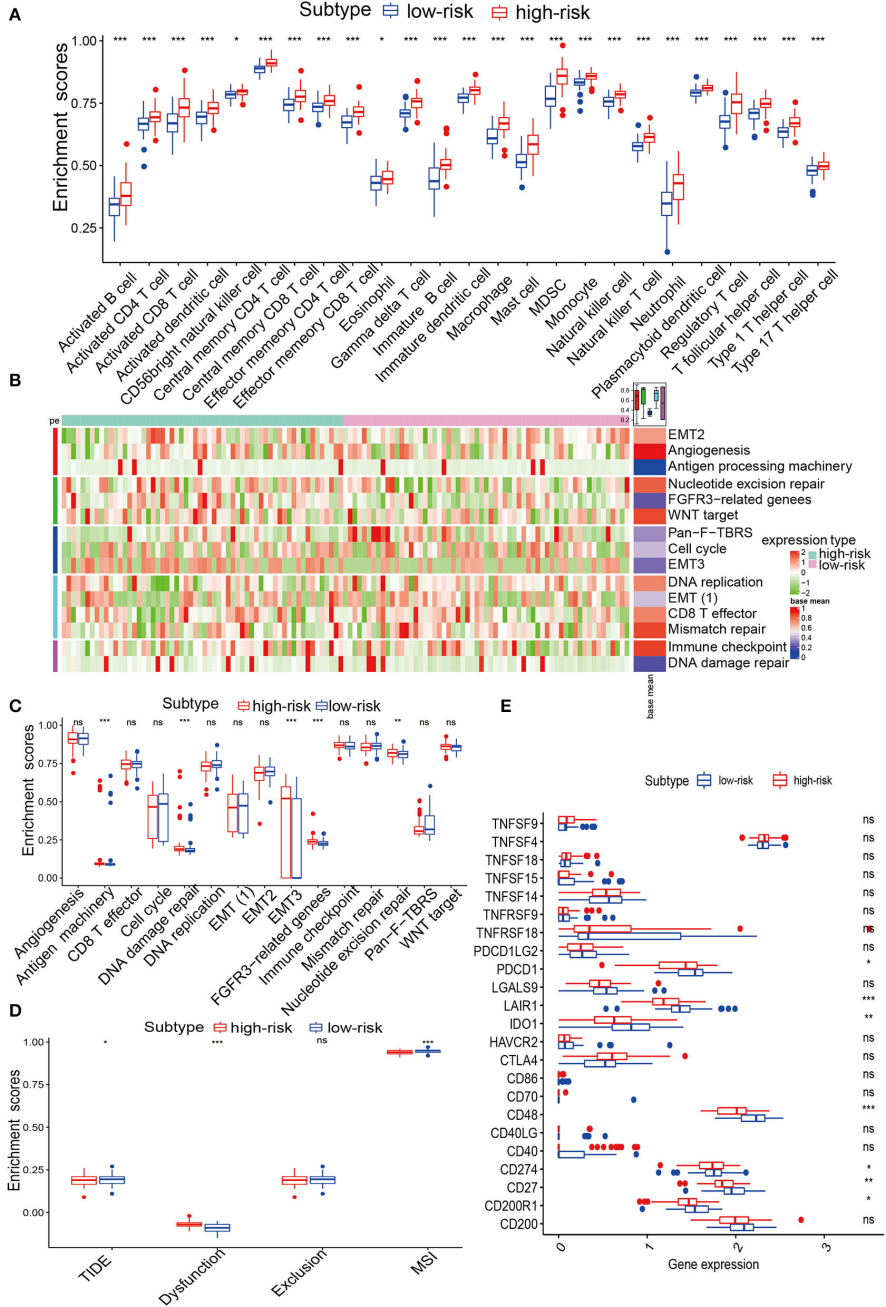
Immune infiltration analysis of IRPM in TCGA. **(A)** Abundance differences in immune cells between both groups in the TCGA-GBM cohort. **(B)** Heatmap showing the distribution of immune pathways in both groups. **(C)** The abundance of distinction in immune pathways between both groups in the TCGA-GBM cohort. **(D)** TIDE, MSI, and T cell exclusion and dysfunction score in IRPM. Comparison of scores between IRPM by Wilcoxon test. **(E)** Quantification of immune checkpoints in both groups in the TCGA-GBM cohort.

To assess the immune status of IRPM, ssGSEA was used to analyse the immune infiltration of GBM samples. We used heatmaps to determine the distribution of 28 immune cells in the TCGA and CGGA cohorts and quantitatively analyzed 28 immune cells. The results revealed that 28 immune cells were enriched in the high-risk group (Figure 5A and Supplementary Figures S6A–C), which suggested that patients with immune cell enrichment might have an immune emergency status in the high-risk group. To investigate the various characteristics of immune pathways in both groups, typical biological processes were employed to compare potential underlying mechanisms. A heatmap analysis of immune-related signatures was performed for the high and low-risk GBM samples (Figure 5B). Further quantitative analysis of the immune characteristics of the different subgroups showed that antigen processing machinery, DNA damage repair, epithelial-mesenchymal transition (EMT)3, fibroblast growth factor receptor (FGFR)3, and nucleotide were significantly enriched in the high-risk group in the TCGA cohort (Figure 5C), and CD8 T effector, EMT(1), and EMT(2) were significantly enriched in the high-risk group in the CGGA cohort (Supplementary Figure S6D). Next, we analyzed the immune checkpoints in the two groups and found that *CD200R1, CD27, CD274* (*PD-L1*), *CD48, CTLA4, IDO1, LAIR1*, and *PDCD1* were dramatically higher in the high-risk group in the TCGA cohort (Figure 5E), and all 22 immune checkpoints were remarkably higher in a high-risk group for CGGA cohort (Figure S6E). To determine the relevance between IRPM and immune types (C1–C6), we further explored the distribution of the six pan-cancer immune types in IRPM and found that the proportion of C3 was greater in the low-risk group than in the high-risk group. Moreover, the proportion of C1 and C2 was elevated, which could also explain the different survival times between the two groups (Supplementary Figure S6F). The TIDE is associated with a greater likelihood of immune evasion, suggesting that patients are less likely to benefit from ICB treatment. In our results, the high-risk group had a lower TIDE score than the low-risk group, implying that high-risk patients could benefit more from ICB therapy than low-risk patients (Figure 5D). To determine the advantages of riskscore, the AUC of riskscore was better at 1 and 3 years than TIDE and TIS (Supplementary Figures S7A,B). Therefore, The performance of risk scores was higher than TIDE and TIS. To thoroughly analyse the relationship between IRPM and immunotherapy, we retrieved the GSE78220 cohort and analyzed IRPM for anti-PD-1 validation. The patients in the response group and the *BRCA2* mutation group were mainly concentrated in the low riskscore group (Supplementary Figures S7C,D). Survival analysis of patients receiving pembrolizumab showed that there was notably lower survival in the high-risk group than in the low-risk group (Supplementary Figure S7E). We revealed that the riskscore of partial response in the responding group was notably lower than that of partial response (Supplementary Table S5, Supplementary Figure S7F). Our IRPM could help clinicians identify patients who are sensitive to PD-1 immune checkpoint blockade. Finally, we quantitatively analyzed the levels of the Estimatescore, Stromalscore, and Immunescore between the two groups, and found significantly higher scores in the high-risk group than in the low-risk group (Supplementary Figures S8A–C). This result further revealed the conspicuous distinction in immune status between both subgroups in IRPM. The radar map showed a remarkable association between the expression value of PD-L1 and riskscore, again confirming the ability of the scoring system to accurately predict the outcome of immunotherapy (Supplementary Figure S8D). By analysis of *CD274*, the association between the biomarker and response was reversed in several cancer types. This phenomenon might be due to the heterogeneity among cancers in terms of immune infiltration. The above results displayed that there was a considerable distinction between the immunotherapy of both groups. Thus, IRPM could predict the response to immunotherapy for glioma.

### Comparisons of Somatic Mutations in High and Low-Risk Groups

**Figure 6:**
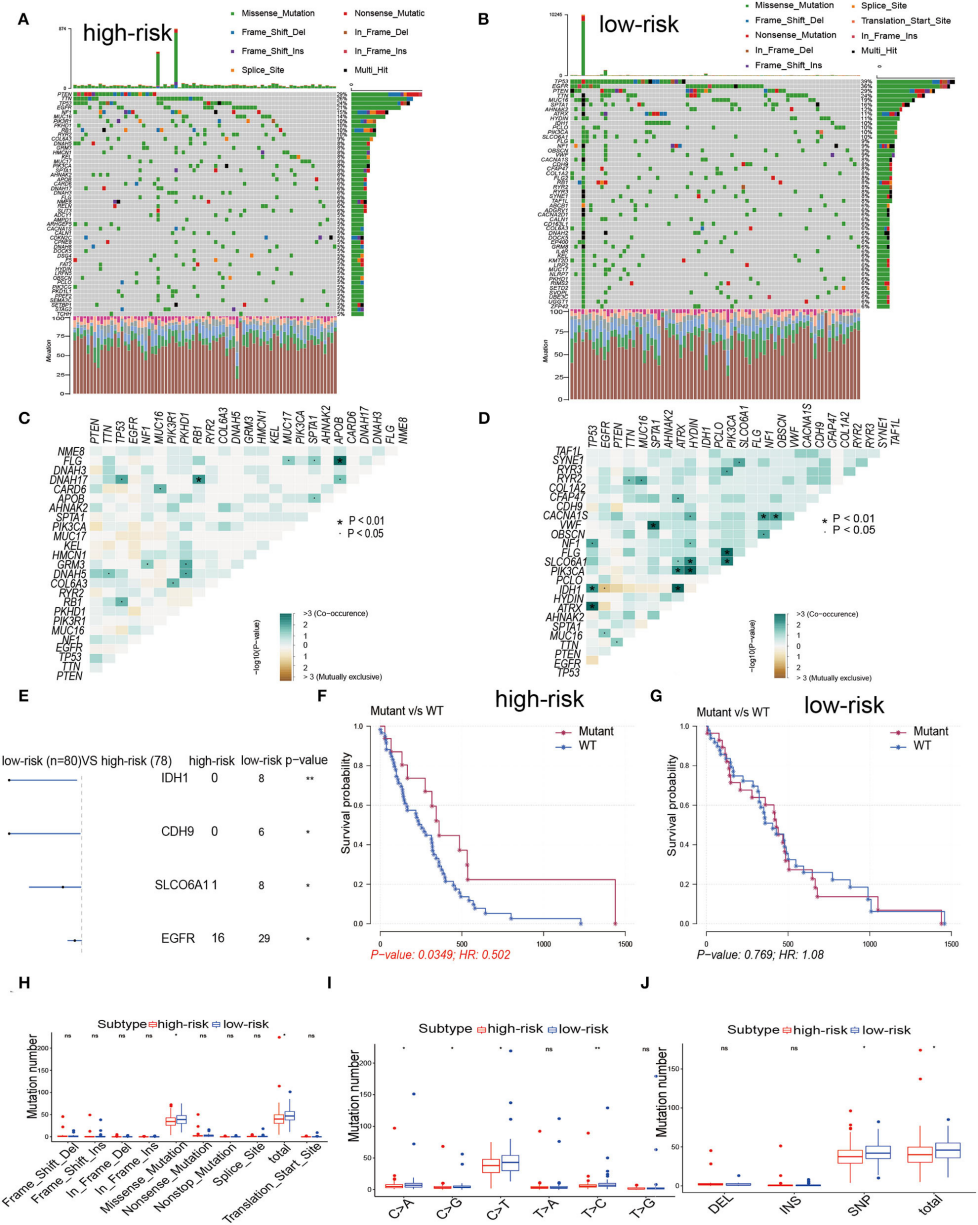
The landscape of somatic Mutations in high and low-risk groups. **(A**,**B)** The distribution of mutations based on the top 50 most commonly mutated genes is displayed in the waterfall plot. Each GBM sample mutation type is shown in the central panel, and the TMB for each GBM sample is shown in the upper panel. The mutated genes and mutation rate of genes mutated in both cohorts are shown. The lower section shows the SNV type for each sample. The top panel showed the tumor mutational burden. **(C,D)** The heatmap analyses of the mutual co-occurrence and exclusion mutations based on the top 25 commonly mutated genes. Each cell color and symbol represents the statistical significance of exclusivity or co-occurrence of each gene pair. **(E)** The forest plot reveals the top 4 most distinctively mutated genes between the two cohorts. **(F,G)** KM curves reveal the independence between OS and EGFR mutations in high and low-risk cohorts. **(H)** Each mutation type is categorized by effects, INDEL, SNP **(I)**, and SNV **(J)**.

For the above outcomes, we concluded that patients in both groups were associated with corresponding immune infiltration status. Related literature implies that immune infiltration status may also be associated with mutation (5, 26, 27). To investigate whether immune infiltration status was associated with mutation rates, we performed mutation rate analysis for both groups. In the low-risk group, more than 6% of the samples had mutations in 50 genes, while in the high-risk group only 27 genes met this criterion, 15 genes of which overlapped. The 50 genes with the highest mutation frequencies in the corresponding groups were indicated in Figures 6A,B. Interestingly, the mutation rates of PTEN, TTN, EGFR, NF1, TP53, MUC16, and SPTA1 in both groups were higher than 15%, and there were interactions among them to manage multiple tumor-related biological processes in GBM (Figures 6A,B), which indicated that they might be primarily involved in tumor progression. Next, the first 25 mutated genes were studied for co-mutations and exclusive mutations using the comet algorithm. Four cases (*IDH1*-*TP53, IDH1*-*ATRX, PIK3CA*-*HYDIN, FLG*-*APOB*) displayed mutually exclusive mutations compared with the pervasive mutually exclusive landscape, suggesting that they might have redundant effects in the same pathway and a selective advantage of retaining a copy of the mutation between them (Figures 6C,D). The incidence of IDH1 mutations was significantly higher in the low-risk group compared to the high-risk group, which also indicates that patients in the low-risk group have a better prognosis, consistent with the results of the previous studies in this study (Figure 6E). Furthermore, *EGFR* was another typical example demonstrating a possible chain reaction of different mutation sites between the two groups (Figures 6F,G). After detecting the above RNA_seq alterations, we further explored whether there was evidence of the distinction between both groups at the genomic level. The R package “maftools” was used to analyse and visualize somatic mutations, including SNV, SNP, INS, and DEL. Because the majority of genomic variants in the two cohorts were missense mutations (80%). Hence, it was imperative to quantify the mutation types and reveal their potential significance (Figure 6H). As for SNVs, all sample populations were studied, with C > T being the most common type in both groups. For most types of SNV, the mutation number was significantly higher in the low-risk group than in the high-risk group (Figure 6I). Additionally, we uncover that the number of SNP in the low-risk cohort exceeded that in the high-risk cohort. Although the number of the four kinds of somatic mutations differed significantly between the two groups, the internal composition ratio of every mutation type among all variants remained nearly constant, suggesting that the observed distinction in the number of mutations was not caused by a type switch (Figure 6J). Figure 6E showed that the mutation rate of *IDH1* was greater in the low-risk group than in the high-risk group, and these mutations predicted a good prognosis in the early studies (28, 29). We revealed that the higher number of mutations in the low-risk group might be predictive of a better consequence as compared with the high-risk group, which could explain the poor efficacy of immunotherapy in the low-risk group. Finally, significant copy number amplifications and deletions were detected and compared in both groups with a threshold of FDR < 0.05. We observed that more regions were altered in the high-risk group (Supplementary Figures S9A,B), while the proportion of amplification in the low-risk group was higher than that in the high-risk group (Supplementary Figures S9C,D). Most of the genes that CNV corresponds in the low-risk cohort were mainly occupied in half of the samples in the cohort, while it even made up one-third of the high-risk cohort (Supplementary Figures S9E,F). By calculating the frequency of each CNV across all patients, we revealed that 7p11.2 and 9p21.3 were the most frequent CNVs in the high-risk group; whereas 7p11.2 and 9p21.3 loss were also among the most common changes that occurred in the low subgroup. Overall, we found similarities between chromosomal aberrations in the two groups, but the AMP and DEL of chromosomal aberration sites were higher in the low-risk group than in the high-risk group. These results of CNV lead to altered expression of the corresponding genes. In combination with the immunotherapy results above, we found higher CNV was positively correlated with clinical benefits from ICB. Considering the results obtained for the mutations, we found a prominent distinction between two groups in these two aspects, implying that IRPM might have a predictive role in mutations.

### Depiction of the DNA Methylation Pattern in GBM

**Figure 7:**
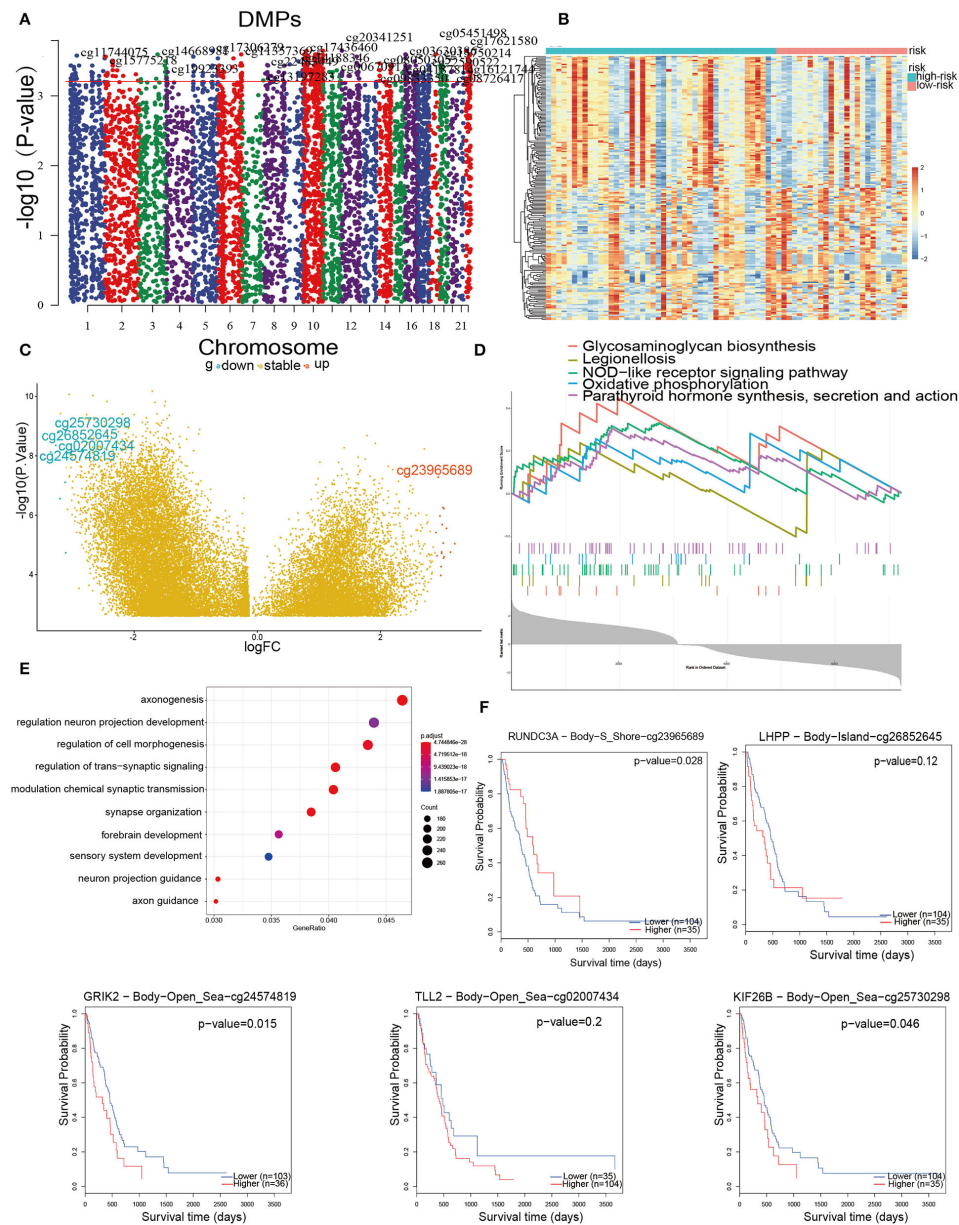
DNA methylation pattern in high and low-risk groups. **(A)** Manhattan mapping of genome-wide DNA methylation differences in both groups. **(B)** Heatmap plot of the DMPs in two groups. **(C)** Volcano plot of DMPs based on both groups; Red illustrates downregulated genes, and blue illustrates upregulated genes. **(D)** GSEA showed considerable enrichment in five biological processes. Genes are listed by Δbeta. **(E)** GO enrichment analysis shows DEGs between both groups. Genes were ranked by Δbeta. **(F)** Kaplan-Meier curves of the five methylation sites with the largest fold difference.

The inability to sustain normal DNA methylation, including high methylation of CpG islands and CpG-poor regions, heightens the sensitivity to trigger tumor initiation and progression ^30^. Hence, a goal was to use Illumina Infinium 450 k DNA methylation data to explore and contrast the influence of DNA methylation patterns in IRPM. In this section, DMPs were performed using ChAMP on 169 samples that had no more than 20% of genes with a missing beta value. Taking the criteria of ΔDbeta >0.15 and FDR < 0.01, a total of 7,225 immune-associated DMPs were detected (Figure 7A). These DMPs were further visualized by heatmap and volcano map (Figures 7B,C). The results revealed that the methylation levels in the high-risk group were significantly higher than those in the low-risk group. We obtained the five DMPs with the largest logFC, which were cg25730298, cg26852645, cg02007434, cg24574819, cg23965689. Cg23965689, cg24574819, and cg25730298 were related to survival (Figure 7F). These three methylation sites could regulate the expression of corresponding genes (*RUNDC3A, GRIK2*, and *KIF26B*) that cause tumor growth and development, and therefore these sites might become new targets for tumor treatment. Finally, these DMPs corresponding to the DEGs were subjected to GO and GSEA enrichment analysis (Figures 7D,E). The GO analysis was mainly enriched in signaling pathways such as axonogenesis, regulation of neuron projection development, and regulation of cell morphogenesis neuron associated biological processes, and GSEA was mainly enriched in glycosaminoglycan biosynthesis, legionellosis, NOD-like receptor signaling pathway, oxidative phosphorylation, parathyroid hormone synthesis, secretion, and action, indicating that aberrant methylation-induced immune aggressive behavior of tumors *via* the recognition and involvement of neural and glycolytic pathways. Combining the above methylation results, IRPM has different methylation levels in response to the prognosis of glioma patients, and cg23965689, cg24574819, and cg25730298 may also be potential targets for the treatment of glioma in the future.

## Discussion

This study includes a multi-omics analysis of immune-related genes to explore the impact of these genes on the survival of GBM patients. We extracted data from TCGA, GTEx, and CGGA, including mRNA expression, mutations, and DNA methylation. Based on our analysis of IRPM, we found its good performance in predicting the prognosis of GBM and analyzed the immune infiltration, mutation, and methylation status of each subgroup which provides more comprehensive support for IRPM to assess GBM patients.

Most immune genes can affect tumor TME. In our study, based on the immune gene dataset, we applied WGCNA and lasso to identify three immune-related hub genes and constructed IRPM based on three independent OS prognostic factors (*IL1R1, VDR*, and *TNFSF12*). *IL1R1* is a cytokine receptor belonging to the interleukin 1 receptor family and is an essential mediator involved in many cytokine-induced immune and inflammatory responses. Tumor cells secrete or receive the inflammatory factor *IL1*, which is closely associated with the prognosis of malignant tumor development, invasion, metastasis, and chemotherapy resistance (30). The protein encoded by *TNFSF12* is a cytokine that belongs to the tumor necrosis factor (*TNF*) ligand family. It is a binding agent for the FN14/TWEAKR receptor. By promoting the proliferation and migration of endothelial cells, this cytokine has been found to play a role in regulating angiogenesis (31, 32). *VDR* encodes the nuclear hormone receptor for vitamin D3, which is also the second receptor for cholecalciferol. The regulation of gene expression by this protein is mainly through a series of metabolic response pathways, including immune response and tumor activation pathways (33). Above all, these three immune genes have a role in promoting tumor proliferation, immunity, and invasion.

To further understand the immunological properties of IRPM, then we investigated immune infiltration in different subgroups. The concept of immunotherapy in neuro-oncology has been developed for decades but was mainly hampered by poorly defined relevant antigens and selective targets in the central nervous system. Checkpoint inhibitors and vaccines have recently achieved remarkable success in clinical immuno-oncology (34–36). ICBs have been shown to be an attractive therapy for patients with recurrent or refractory tumors.

Firstly, knowledge of the tumor immune microenvironment can help to determine new ways to treat GBM and improve the efficacy of immunotherapy. In combination with other molecular and immune subtypes, IRPM could identify different molecular and immune subtypes of GBM. According to the immune subtype classification of GBM, patients with the immune active subtype in the high-risk group comprised the major component while the immune depleted subtype in the low-risk group comprised the minor part. In previous studies, immune active and immune depleted subtypes showed significant differences in M1/M2 macrophages, B cells, and cytolytic activity, but not in T cells, CD8 T+ cells, and cytotoxic cells. Immunoreactive subtypes are closely related to immunoreactive pathways and gene sets, and immunodepleted subtypes are characterized by tumor-promoting signals, to suppress host immune responses, such as activation of the Wnt/transforming growth factor-β signaling pathway (37). Hence, patients in the high-risk group might have a stronger immune response to tumorigenesis and tumor progression and consequently benefit from ICB therapy than patients in a low-risk group.

Immune checkpoint blockade therapy has been proven to be an efficient therapy for patients with relapsed or refractory GBM (38). Considering that the overall response rate to ICB therapy is still low, it is critical to determine which patients could benefit most from these treatments. Therefore, we performed PD-1 validation of IRPM. In anti-PD-1 patients, the survival in the high-risk group was notably lower than that in the low-risk group, and we found IRPM could differentiate distinct outcomes in patients treated with anti-PD-1 treatment. Studies have shown that targeted therapy with PD-1 significantly improves the survival of GBM patients (39). Taken together, our study firmly indicates that the IRPM was substantially associated with response to anti-PD-1 immunotherapy. IRPM may contribute to clinicians recognizing patients who are more appropriate for immunotherapy.

Considering its significant modulatory effects in EMT and tumor immunosuppression, the integration of conventional therapeutic modalities and TGF-β inhibition has enormous prospects for enhancing the antitumor activity in tumors. Therapeutic strategies against TGF-β contain neutralizing antibodies targeting ligands and receptors, small-molecule inhibitors, and antisense oligonucleotides (40, 41). Galunisertib, which is a small molecule inhibitor of TGF-β receptor I kinas, is being used in clinical trials in collaboration with Nivolumab (PD-1 monoclonal antibody, mAb) in the liver cancer, metastatic PDAC, and NSCLC (NCT02734160, NCT02423343). A previous preclinical investigation indicated that galunisertib in association with anti-PD-L1 treatment had remarkably superior tumor abrogation and anti-tumor efficacy than either galunisertib or anti-PD-L1 monotherapy, suggesting that galunisertib induces increased anti-tumor T cell immunity (42). Our results showed a higher EMT3 enrichment and a lower survival advantage in the high-risk group, which is in accordance with the above findings and further demonstrates the accuracy of IRPM.

The biomarkers, such as TIDE and TIS, have been reported to forecast patient response to immunotherapy. TIDE is a creative computational approach to identifying two mechanisms of tumor immune evasion: inducing T-cell dysfunction in tumors with high levels of cytotoxic T lymphocytes (CTL) and preventing T-cell infiltration in tumors with low levels of CTL (43). In addition, NanoString Technologies developed TIS as a clinical-level assay to provide quantitative and qualitative information about TME, an 18-gene signature that includes genes reflecting sustained adaptive Th1 and cytotoxic CD8 T-cell responses. In forecasting reaction to anti-PD-1/PD-L1 therapy, TIS showed favorable results and has been validated in the many tumor cohorts treated with mono-pembrolizumab, showing a positive correlation with response and survival (44). However, neither TIDE nor TIS focus on the function and status of T cells and do not completely reflect the complexity of TME involvement in the immunotherapeutic response. Furthermore, TIDE and TIS focus on patient response to immunotherapy rather than patient survival time, and life expectancy is also important in treatment decisions. In our study, the predictive value of IRPM riskscore was comparable to that of TIDE and TIS, and IRPM might be a better predictor of long-term OS follow-up. In addition, IRPM consists of only three genes and is easier to detect than TIDE and TIS, and it may be a better predictor of OS at longer follow-up.

Secondly, to further understand the immunological properties of IRPMs based on biological insights, we subsequently investigated gene mutations in different IRPMs. The missense variant was the most frequent, together with nonsense and frame_shift_deletions, as previously reported. Among the largest mutational distinction between the two groups were *IDH1* mutations, which were more prevalent in samples from the low-risk group than in those from the high-risk group (11 vs. 0%). *IDH1* mutations are not only the common single genetic incidents in cancer but are also associated with more invasive diseases and better patient prognosis in numerous cancers, particularly GBM. In 2008, Parsons DW identified *IDH1* mutations for the first time in an exome sequencing study of GBM (45). The finding of this new biological molecular marker provides an essential reference for the treatment and prognosis of glioma patients and may become a new target for future treatment. Therefore, low-risk patients with a high *IDH1* mutation rate have a better prognosis than high-risk patients with a low *IDH1* mutation rate, in agreement with our survival results. Numerous studies in the literature have found that GBM patients with *EGFR* mutations have a worse prognosis than those without mutations (46). In our study, there was a prominent survival difference between the mutation and non-mutation EGFR gene samples in the high-risk group (*p* < 0.05). The highest mutation rate was found in the high-risk group for the *PTEN* gene, a tumor immune tolerance mechanism, which is also known as *MMAC1* and *TEP1*. Current research has found that *PTEN* gene abnormity can exist in many tumors such as glioblastoma, prostate cancer, endometrial cancer, etc. (47). *PTEN* is considered to be another oncogene that is more widely altered and closely related to tumorigenesis than the *p53* gene (48). By analyzing the mutation type and CNV, we revealed that the high mutation numbers in the low-risk group might be accompanied by low immune morphology, these findings may require further study in the future.

Finally, there has been increasing interest in altered DNA methylation in recent years. As altered DNA methylation patterns are a hallmark of tumors, differential methylation of CpG sites has been linked to the expression of genes known to be important in cancer biology (49). Some studies have suggested that unmethylated promoters may be converted to densely methylated forms, like tumor suppressors, which would promote gene silencing. Other sequences may change to hypomethylated forms in tumors, leading to abnormal activation of genes normally repressed by DNA methylation (50). Here, we performed DMPs between the high and low-risk groups, and the heatmap results implied that high-risk patients more frequently showed hypermethylation. The volcano map results showed five CPG sites with the largest differential fold (51). The genes corresponding to these three CPG sites may represent new targets for the treatment of GBM. Based on these findings, we can speculate that high methylation may be associated with the immune status of GBM patients, a hypothesis that must be confirmed in future studies.

We also found some similar reports (52), but to my knowledge, compared to previous work, we first chose to filter immune-related signatures from ImmPort and InnateDB sites in GBM, and got new immune-related signatures, while we also validated the screened genes by immunohistochemistry, and the mutual validation of the database and the molecular experiments made the article more convincing. Finally, we performed a comprehensive comparison of immune infiltration, mutational status, and methylation of IRPM. These are the novel points of our article.

Despite a more compositive knowledge of the tumor immune microenvironment of GBM and a robust predictive model in this study, two major shortcomings require further investigation. The first weakness is that because of the need to match multi-omics and clinical information, we were restricted to data from the TCGA database and could not overlay other data sources. Thus, our ability to detect the reliability of the model was hindered when combined with other data. The second disadvantage is that the employment of predictive models requires three types of histological data, including RNA_seq, mutation, and DNA methylation data, which is cost-intensive and not easy to implement in practical applications. Nevertheless, the accelerated development of biotechnology promises to produce a trinity of toolkits that will pave the way for their implementation and generalization. Despite such limitations, it is undeniable that our study provides a better prognostic model for GBM. Furthermore, IRPM may show compelling clinical value, which may enhance the overall survival of GBM patients and even lead to the development of new treatment strategies for GBM patients.

## Conclusions

In conclusion, we conducted an in-depth multi-omics exploration of IRPM, and also performed multi-omics to assess IRPM, which could evaluate the response to immunotherapy and enhance the accuracy of predicting the prognosis. IRPM gives us hope that we may soon have the tools and knowledge needed to use these models as weapons in the fight against cancer.

## Data Availability Statement

The original contributions presented in the study are included in the article/Supplementary Material, further inquiries can be directed to the corresponding author/s.

## Ethics Statement

The studies involving human participants and human specimens were approved by the Ethics Committee of the Second Hospital of Harbin Medical University with the reference number KY2021-172. All methods were carried out in accordance with relevant guidelines and regulations. The patients/participants provided their written informed consent to participate in this study. Written informed consent was obtained from the individual(s) for the publication of any potentially identifiable images or data included in this article.

## Author Contributions

SM, JD, and SH conceived and designed the study and drafted the manuscript. SM, FW, NW, JJ, YB, and HJ provided analytical technical support. All authors have read and approved the final manuscript.

## Funding

This work was funded by the National Natural Science Foundation of China (No. 61575058).

## Conflict of Interest

The authors declare that the research was conducted in the absence of any commercial or financial relationships that could be construed as a potential conflict of interest.

## Publisher's Note

All claims expressed in this article are solely those of the authors and do not necessarily represent those of their affiliated organizations, or those of the publisher, the editors and the reviewers. Any product that may be evaluated in this article, or claim that may be made by its manufacturer, is not guaranteed or endorsed by the publisher.

## References

[1] Alcantara Llaguno SR Chen J Parada LF. Signaling in malignant astrocytomas: role of neural stem cells and its therapeutic implications. Clin Cancer Res. (2009) 15:7124–9. 10.1158/1078-0432.CCR-09-0433 19934302 PMC2787668

[2] Chen Y Li ZY Zhou GQ Sun Y. An immune-related gene prognostic index for head and neck squamous cell carcinoma. Clin Cancer Res. (2020) 27:330–41. 10.1158/1078-0432.CCR-20-2166 33097495

[3] Dai Y Qiang W Lin K Gui Y Lan X Wang D. An immune-related gene signature for predicting survival and immunotherapy efficacy in hepatocellular carcinoma. Cancer Immunol Immunother. (2021) 70:967–79. 10.1007/s00262-020-02743-0 33089373 PMC10992402

[4] Topalian SL Taube JM Anders RA Pardoll DM. Mechanism-driven biomarkers to guide immune checkpoint blockade in cancer therapy. Nat Rev Cancer. (2016)16:275–87. 10.1038/nrc.2016.36 27079802 PMC5381938

[5] Ma S Ba Y Ji H Wang F Du J Hu S. Recognition of tumor-associated antigens and immune subtypes in glioma for mRNA vaccine development. Front Immunol. (2021) 12:738435. 10.3389/fimmu.2021.738435 34603319 PMC8484904

[6] Goc J Germain C Vo-Bourgais TK Lupo A Klein C Knockaert S. Dendritic cells in tumor-associated tertiary lymphoid structures signal a Th1 cytotoxic immune contexture and license the positive prognostic value of infiltrating CD8+ T cells. Cancer Res. (2014) 74:705–15. 10.1158/0008-5472.CAN-13-1342 24366885

[7] Gartrell RD Marks DK Rizk EM Bogardus M Gérard CL Barker LW. Validation of Melanoma Immune Profile (MIP), a Prognostic Immune Gene Prediction Score for Stage II–III Melanoma. Clin Cancer Res. (2019) 25:2494–502. 10.1158/1078-0432.Ccr-18-2847 30647081 PMC6594682

[8] Zhang Y Yang M Ng DM Haleem M Yi T Hu S. Multi-omics data analyses construct TME and Identify the immune-related prognosis signatures in human LUAD. Mol Ther Nucleic Acids. (2020) 21:860–73. 10.1016/j.omtn.2020.07.024 32805489 PMC7452010

[9] Johnson WE Li C Rabinovic A. Adjusting batch effects in microarray expression data using empirical Bayes methods. Biostatistics. (2007) 8:118–27. 10.1093/biostatistics/kxj037 16632515

[10] Ayers M Lunceford J Nebozhyn M Murphy E Loboda A Kaufman DR. IFN-gamma-related mRNA profile predicts clinical response to PD-1 blockade. J Clin Invest. (2017) 127:2930–40. 10.1172/JCI91190 28650338 PMC5531419

[11] Yoshihara K Shahmoradgoli M Martinez E Vegesna R Kim H Torres-Garcia W. Inferring tumour purity and stromal and immune cell admixture from expression data. Nat Commun. (2013) 4:2612. 10.1038/ncomms3612 24113773 PMC3826632

[12] Langfelder P Horvath S. WGCNA: an R package for weighted correlation network analysis. BMC Bioinformatics. (2008) 9:559. 10.1186/1471-2105-9-559 19114008 PMC2631488

[13] Yi M Li T Qin S Yu S Chu Q Li A. Identifying tumorigenesis and prognosis-related genes of lung adenocarcinoma: based on weighted gene coexpression network analysis. Biomed Res Int. (2020) 2020:4169691. 10.1155/2020/4169691 32149105 PMC7035528

[14] Yu G Wang LG Han Y He QY. clusterProfiler: an R package for comparing biological themes among gene clusters. OMICS. (2012) 16:284–7. 10.1089/omi.2011.0118 22455463 PMC3339379

[15] Luo Q Vogeli TA. A Methylation-based reclassification of bladder cancer based on immune cell genes. Cancers. (2020)12:3054. 10.3390/cancers12103054 33092083 PMC7593922

[16] Cheng Y Wang K Geng L Sun J Xu W Liu D. Identification of candidate diagnostic and prognostic biomarkers for pancreatic carcinoma. EBioMedicine. (2019) 40:382–93. 10.1016/j.ebiom.2019.01.003 30639415 PMC6412825

[17] Du J Yan X Mi S Li Y Ji H Hou K. Identification of prognostic model and biomarkers for cancer stem cell characteristics in glioblastoma by network analysis of multi-omics data and stemness indices. Front Cell Dev Biol. (2020) 8:558961. 10.3389/fcell.2020.558961 33195193 PMC7604309

[18] Tibshirani R. The lasso method for variable selection in the Cox model. Stat Med. (1997) 16:385–95. 10.1002/(sici)1097-0258(19970228)16:4<385::aid-sim380>3.0.co;2-3 9044528

[19] Wang L Shen J Thall PF. A modified adaptive lasso for identifying interactions in the cox model with the heredity constraint. Stat Probab Lett. (2014) 93:126–33. 10.1016/j.spl.2014.06.024 25071299 PMC4111275

[20] Jia Q Wu W Wang Y Alexander PB Sun C Gong Z. Local mutational diversity drives intratumoral immune heterogeneity in non-small cell lung cancer. Nat Commun. (2018) 9:5361. 10.1038/s41467-018-07767-w 30560866 PMC6299138

[21] Koboldt DC Zhang Q Larson DE Shen D McLellan MD Lin L. VarScan 2: Somatic mutation and copy number alteration discovery in cancer by exome sequencing. Genome Res. (2012) 22:568–76. 10.1101/gr.129684.111 22300766 PMC3290792

[22] Ceccarelli M Barthel FP Malta TM Sabedot TS Salama SR Murray BA. Molecular profiling reveals biologically discrete subsets and pathways of progression in diffuse glioma. Cell. (2016) 164:550–63. 10.1016/j.cell.2015.12.028 26824661 PMC4754110

[23] Verhaak RG Hoadley KA Purdom E Wang V Qi Y Wilkerson MD. Integrated genomic analysis identifies clinically relevant subtypes of glioblastoma characterized by abnormalities in PDGFRA, IDH1, EGFR, and NF1. Cancer Cell. (2010) 17:98–110. 10.1016/j.ccr.2009.12.020 20129251 PMC2818769

[24] Du J Ji H Ma S Jin J Mi S Hou K. m6A regulator-mediated methylation modification patterns and characteristics of immunity and stemness in low-grade glioma. Brief Bioinform. (2021) 22:bbab013. 10.1093/bib/bbab013 33594424

[25] Tamborero D Rubio-Perez C Muinos F Sabarinathan R Piulats JM Muntasell A. A pan-cancer landscape of interactions between solid tumors and infiltrating immune cell populations. Clin Cancer Res. (2018) 24:3717–28. 10.1158/1078-0432.CCR-17-3509 29666300

[26] Huang X Zhang G Tang T Liang T. Identification of tumor antigens and immune subtypes of pancreatic adenocarcinoma for mRNA vaccine development. Mol Cancer. (2021) 20:44. 10.1186/s12943-021-01310-0 33648511 PMC7917175

[27] Rooney MS Shukla SA Wu CJ Getz G Hacohen N. Molecular and genetic properties of tumors associated with local immune cytolytic activity. Cell. (2015) 160:48–61. 10.1016/j.cell.2014.12.033 25594174 PMC4856474

[28] Yang P Cai J Yan W Zhang W Wang Y. Classification based on mutations of TERT promoter and IDH characterizes subtypes in grade II/III gliomas. Neuro Oncol. (2016) 18:1099–1108. 10.1093/neuonc/now021 26957363 PMC4933482

[29] Turcan S Rohle D Goenka A Walsh LA Fang F Yilmaz E. IDH1 mutation is sufficient to establish the glioma hypermethylator phenotype. Nature. (2012) 483:479–83. 10.1038/nature10866 22343889 PMC3351699

[30] Boraschi D Italiani P Weil S Martin MU. The family of the interleukin-1 receptors. Immunol Rev. (2018) 281:197–232 10.1111/imr.12606 29248002

[31] Dostert C Grusdat M Letellier E Brenner D. The TNF family of ligands and receptors: communication modules in the immune system and beyond. Physiol Rev. (2019) 99:115–60 10.1152/physrev.00045.2017 30354964

[32] Collette Y Gilles A Pontarotti P Olive D. A co-evolution perspective of the TNFSF and TNFRSF families in the immune system. Trends Immunol. (2003) 24:387–94. 10.1016/s1471-4906(03)00166-2 12860530

[33] Christakos S Dhawan P Verstuyf A Verlinden L Carmeliet GJPR. Vitamin D: metabolism, molecular mechanism of action, and pleiotropic effects. (2016) 96:365. 10.1152/physrev.00014.2015 26681795 PMC4839493

[34] Lim M Xia Y Bettegowda C Weller M. Current state of immunotherapy for glioblastoma. Nat Rev Clin Oncol. (2018) 15:422–42. 10.1038/s41571-018-0003-5 29643471

[35] Burtness B Harrington KJ Greil R Soulières D Tahara M de Castro G. Pembrolizumab alone or with chemotherapy vs. cetuximab with chemotherapy for recurrent or metastatic squamous cell carcinoma of the head and neck (KEYNOTE-048): a randomised, open-label, phase 3 study. The Lancet. (2019) 394:1915–28. 10.1016/s0140-6736(19)32591-7 31679945

[36] Ferris RL Blumenschein G Jr Fayette J Guigay J Colevas AD Licitra L. Nivolumab for recurrent squamous-cell carcinoma of the head and neck. N Engl J Med. (2016) 375:1856–67. 10.1056/NEJMoa1602252 27718784 PMC5564292

[37] Chen YP Wang YQ Lv JW Li YQ Chua MLK Le QT. Identification and validation of novel microenvironment-based immune molecular subgroups of head and neck squamous cell carcinoma: implications for immunotherapy. Ann Oncol. (2019) 30:68–75. 10.1093/annonc/mdy470 30407504

[38] Zhao J Chen AX Gartrell RD Silverman AM Aparicio L Chu T. Immune and genomic correlates of response to anti-PD-1 immunotherapy in glioblastoma. Nat Med. (2019) 25:462–9. 10.1038/s41591-019-0349-y 30742119 PMC6810613

[39] Abril-Rodriguez G Ribas A. SnapShot: immune checkpoint inhibitors. Cancer Cell. (2017) 31:848. 10.1016/j.ccell.2017.05.010 28609660

[40] Colak S Ten Dijke P. Targeting TGF-beta signaling in cancer. Trends Cancer. (2017) 3:56–71. 10.1016/j.trecan.2016.11.008 28718426

[41] Akhurst RJ Hata A. Targeting the TGFbeta signalling pathway in disease. Nat Rev Drug Discov. (2012) 11:790–811 10.1038/nrd3810 23000686 PMC3520610

[42] Holmgaard RB Schaer DA Li Y Castaneda SP Murphy MY Xu X. Targeting the TGFbeta pathway with galunisertib, a TGFbetaRI small molecule inhibitor, promotes anti-tumor immunity leading to durable, complete responses, as monotherapy and in combination with checkpoint blockade. J Immunother Cancer. (2018) 6:47. 10.1186/s40425-018-0356-4 29866156 PMC5987416

[43] Jiang P Gu S Pan D Fu J Sahu A Hu X. Signatures of T cell dysfunction and exclusion predict cancer immunotherapy response. Nat Med. (2018) 24:1550–8. 10.1038/s41591-018-0136-1 30127393 PMC6487502

[44] Seiwert TY Burtness B Weiss J Eder JP Yearley J Murphy E et al. Inflamed-phenotype gene expression signatures to predict benefit from the anti-PD-1 antibody pembrolizumab in PD-L1+ head and neck cancer patients. J Clin Oncol. (2015) 33:6017. 10.1200/jco.2015.33.15_suppl.6017

[45] Eckel-Passow JE Lachance DH Molinaro AM Walsh KM Decker PA Sicotte H. Glioma groups based on 1p/19q, IDH, and TERT Promoter Mutations in Tumors. N Engl J Med. (2015) 372:2499–508 10.1056/NEJMoa1407279 26061753 PMC4489704

[46] Eskilsson E Rosland GV Solecki G Wang Q Harter PN Graziani G. EGFR heterogeneity and implications for therapeutic intervention in glioblastoma. Neuro Oncol. (2018) 20:743–52. 10.1093/neuonc/nox191 29040782 PMC5961011

[47] Jamaspishvili T Berman DM Ross AE Scher HI De Marzo AM Squire JA. Clinical implications of PTEN loss in prostate cancer. Nat Rev Urol. (2018) 15:222–34 10.1038/nrurol.2018.9 29460925 PMC7472658

[48] Zheng H Ying H Yan H Kimmelman AC Hiller DJ Chen AJ. p53 and Pten control neural and glioma stem/progenitor cell renewal and differentiation. Nature. (2008) 455:1129–33 10.1038/nature07443 18948956 PMC4051433

[49] Koch A Joosten SC Feng Z de Ruijter TC Draht MX Melotte V. Analysis of DNA methylation in cancer: location revisited. Nat Rev Clin Oncol. (2018) 15:459–66. 10.1038/s41571-018-0004-4 29666440

[50] Hao X Luo H Krawczyk M Wei W Wang W Wang J. DNA methylation markers for diagnosis and prognosis of common cancers. Proc Natl Acad Sci USA. (2017) 114:7414–9 10.1073/pnas.1703577114 28652331 PMC5514741

[51] Shukla S Pia Patric IR Thinagararjan S Srinivasan S Mondal B Hegde AS. A DNA methylation prognostic signature of glioblastoma: identification of NPTX2-PTEN-NF-kappaB nexus. Cancer Res. (2013) 73:6563–6573 10.1158/0008-5472.CAN-13-0298 24078801

[52] Yi M Li A Zhou L Chu Q Luo S Wu K. Immune signature-based risk stratification and prediction of immune checkpoint inhibitor's efficacy for lung adenocarcinoma. Cancer Immunol Immunother. (2021) 70:1705–19 10.1007/s00262-020-02817-z 33386920 PMC8139885

