# Supplementary material for: Multiomics Data Analysis and Identification of Immune-Related Prognostic Signatures With Potential Implications in Prognosis and Immune Checkpoint Blockade Therapy of Glioblastoma
Source: Front Neurol. 2022 May 20;13:886913. doi: 10.3389/fneur.2022.886913 (PMC9165649; doi:10.3389/fneur.2022.886913)
Supplement: Supplementary file 1 [file Table_1.DOCX]

## Supplemental information

## Supplementary Figures


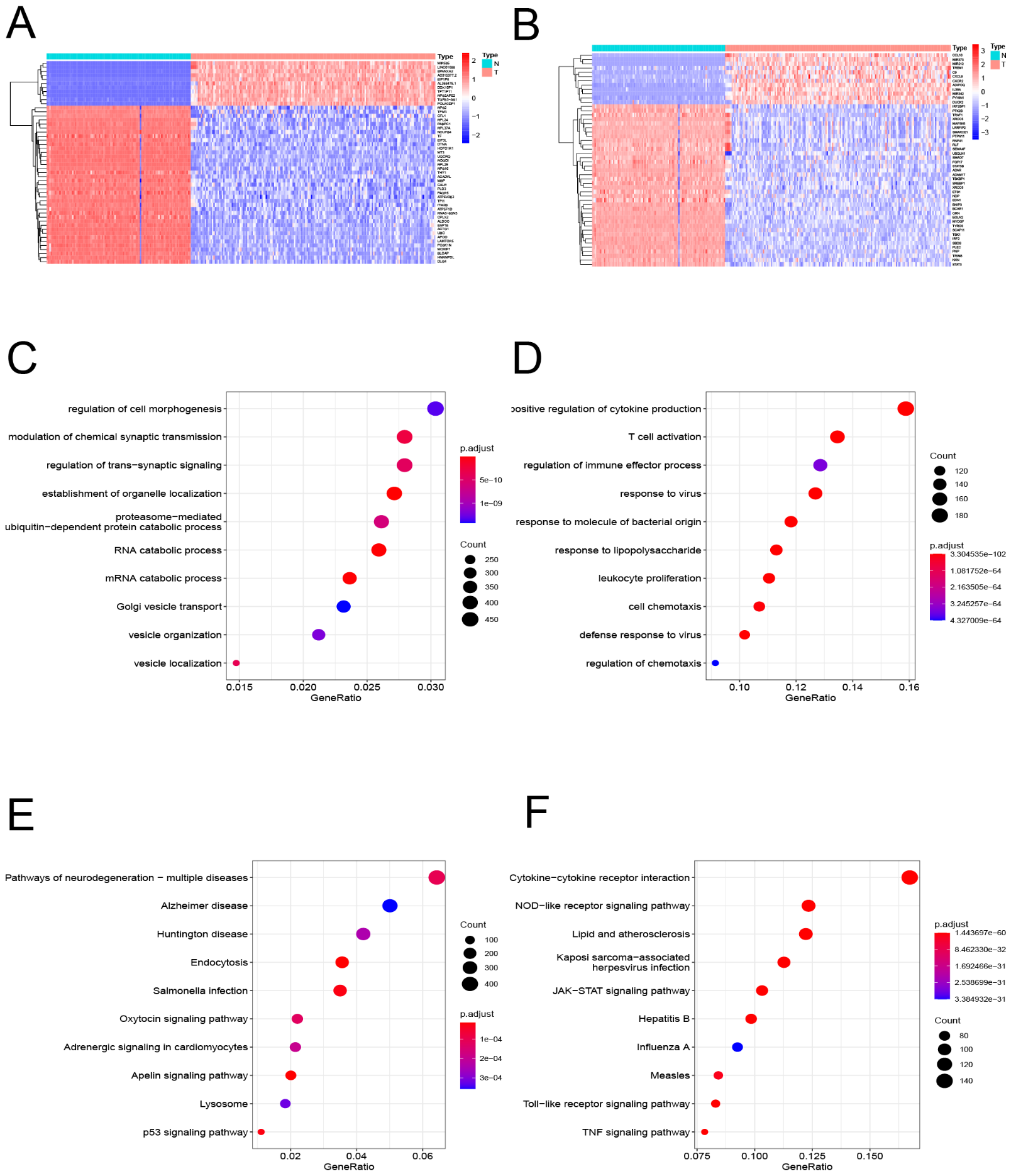


**Figure S1.** Differentially expressed immune‐related genes in GBM.

**(A)**Heatmap displaying all differentially expressed genes (DEGs) between 174 GBM samples (red) and 100 normal samples(green) (p < 0.05, |log2FC| > 2). **(B)** Heatmap displaying immune-related DEGs between 169 GBM samples (red) and 100 normal samples (green). **(C,D)** Gene Ontology (GO) enrichment analysis of normal and tumor DEGs (p< 0.05). **(E,F)** Kyoto Encyclopedia of Genes and Genomes (KEGG) pathway analysis of normal and tumor DEGs (p < 0.05).


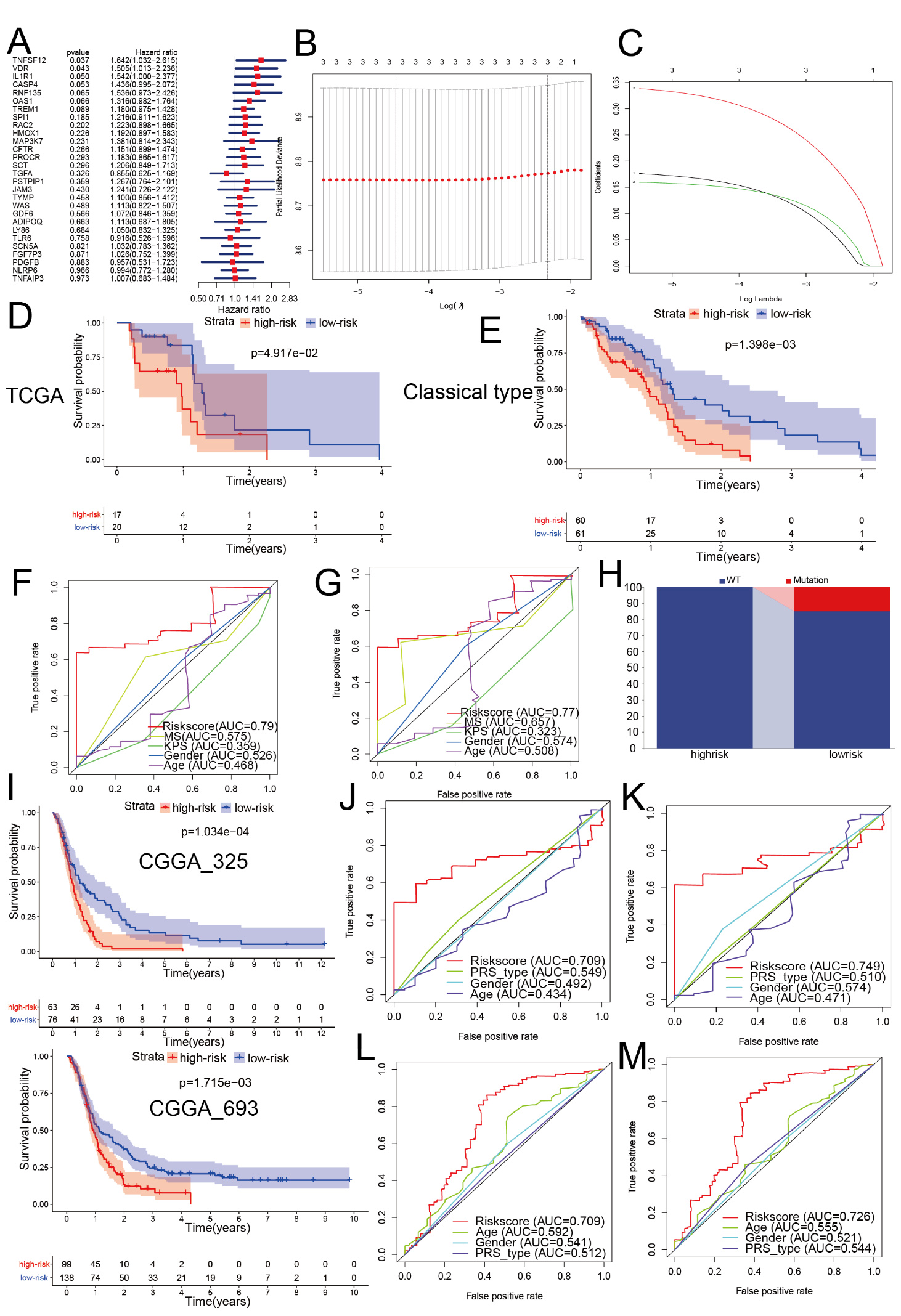


**Figure S2.** Construction of IRPM.

**(A)** Analysis of 28 hub genes for GBM in TCGA by univariate Cox regression. **(B)** Ten-time cross-validation for tuning parameter selection in the TCGA cohort. **(C)** The regression coefficient of identified 3 key genes in the TCGA cohort. **(D, E)** K-M curves displaying the overall survival (OS) of GBM and classical type GBM patients based on the median cutoff value. **(F, G)** ROC curves with calculated AUCs for survival prediction in 3 and 5 years in the TCGA-GBM cohort. **(H, I)** The verified prognostic model with two CGGA databases. **(J-M)** ROC curves with calculated AUCs for risk prediction in 3 year and 5 year in the two CGGA-GBM cohorts. MC, molecular type;


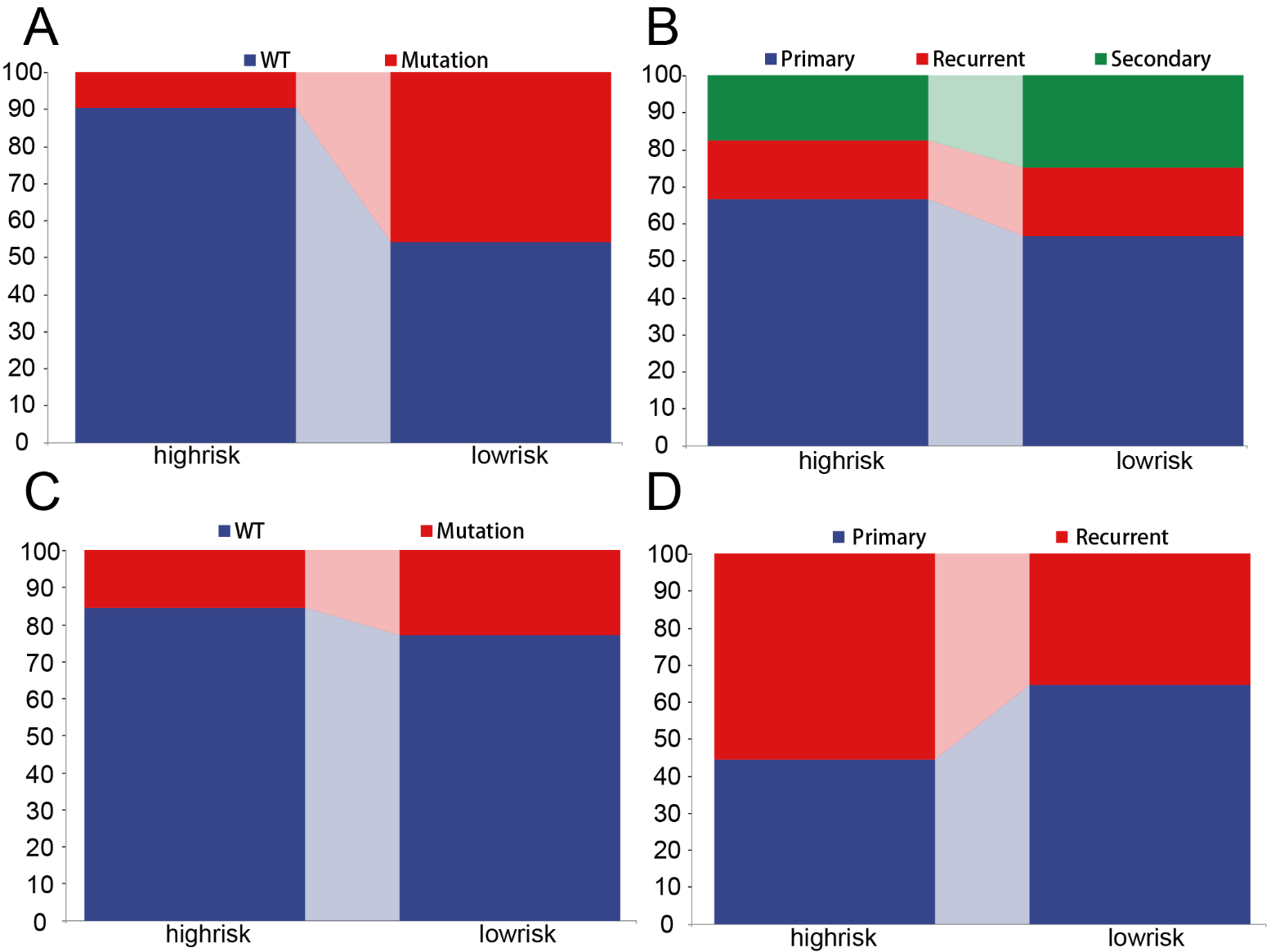


**Figure S3.** The distribution of IDH mutation and recurrence in TCGA and CGGA cohorts.

**(A)** The distribution of IDH mutation in TCGA cohort.

**(B)** The distribution of recurrence in TCGA cohort.

**(C)** The distribution of IDH mutation in CGGA cohort.

**(D)** The distribution of recurrence in CGGA cohort.


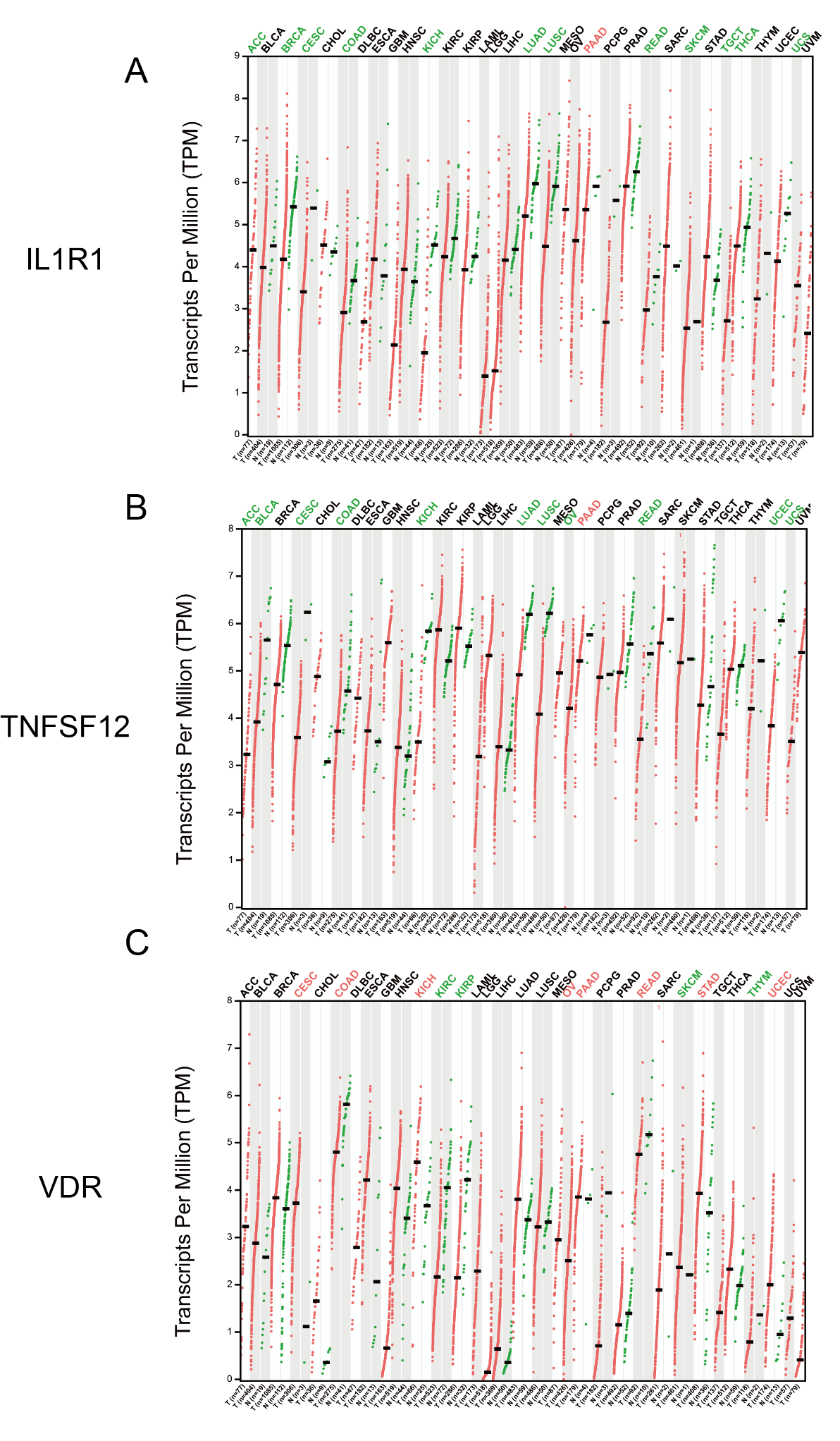


**Figure S4.** Pan-cancer analysis of hub genes.

**(A)** Pan-cancer expression of *IL1R1* genes. **(B)** Pan-cancer expression of *TNFSF12* genes. **(C)** Pan-cancer expression of *VDR* genes.


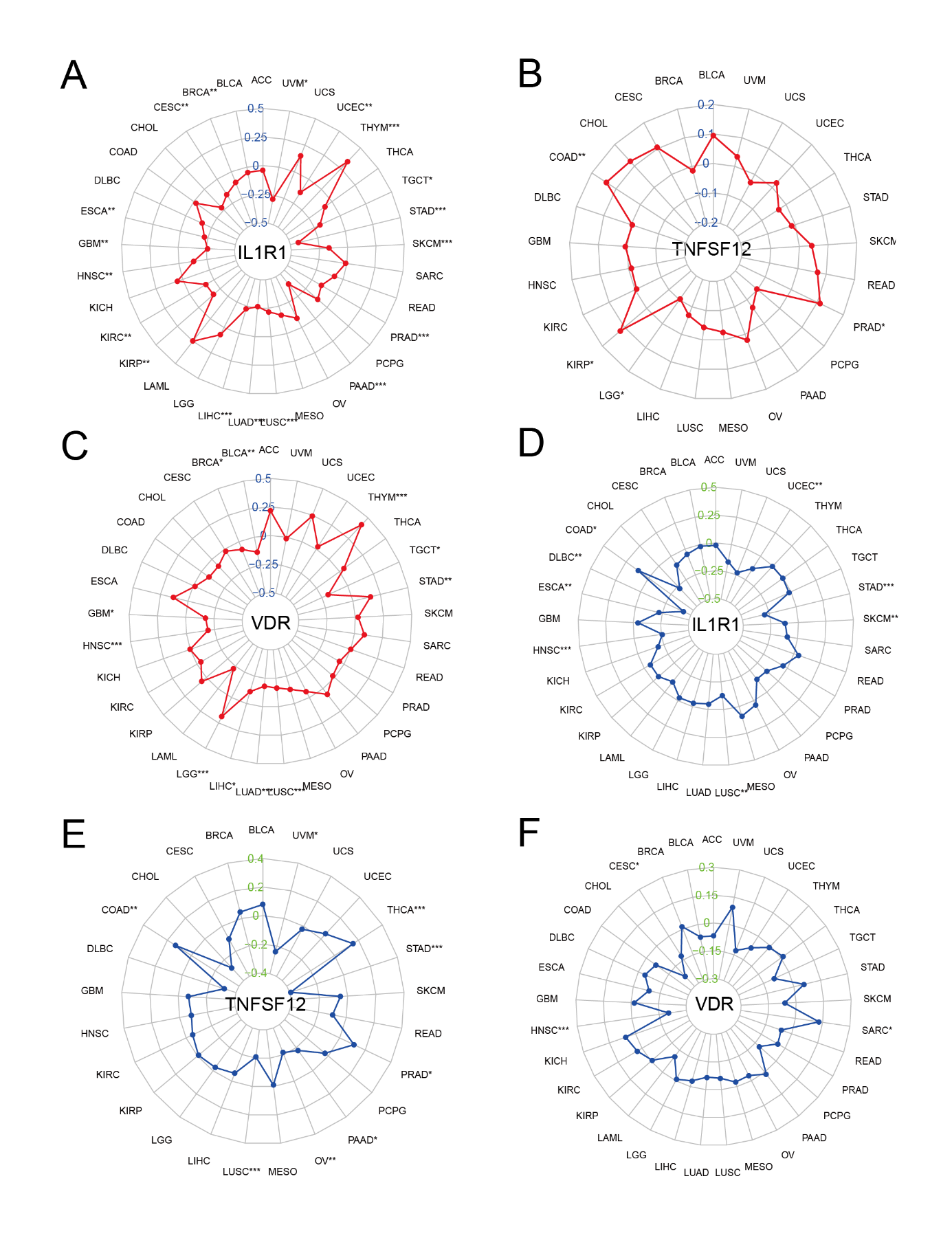


**Figure S5.** TMB, MIS of pan-cancer analysis of hub genes.

**(A-C)** Radar chart of the correlation between hub genes and TMB. **(D-F)** Radar chart of the correlation between hub genes and MSI. The asterisks represented the statistical P-value (∗p <0.05;∗∗p <0.01;∗∗∗p <0.001).


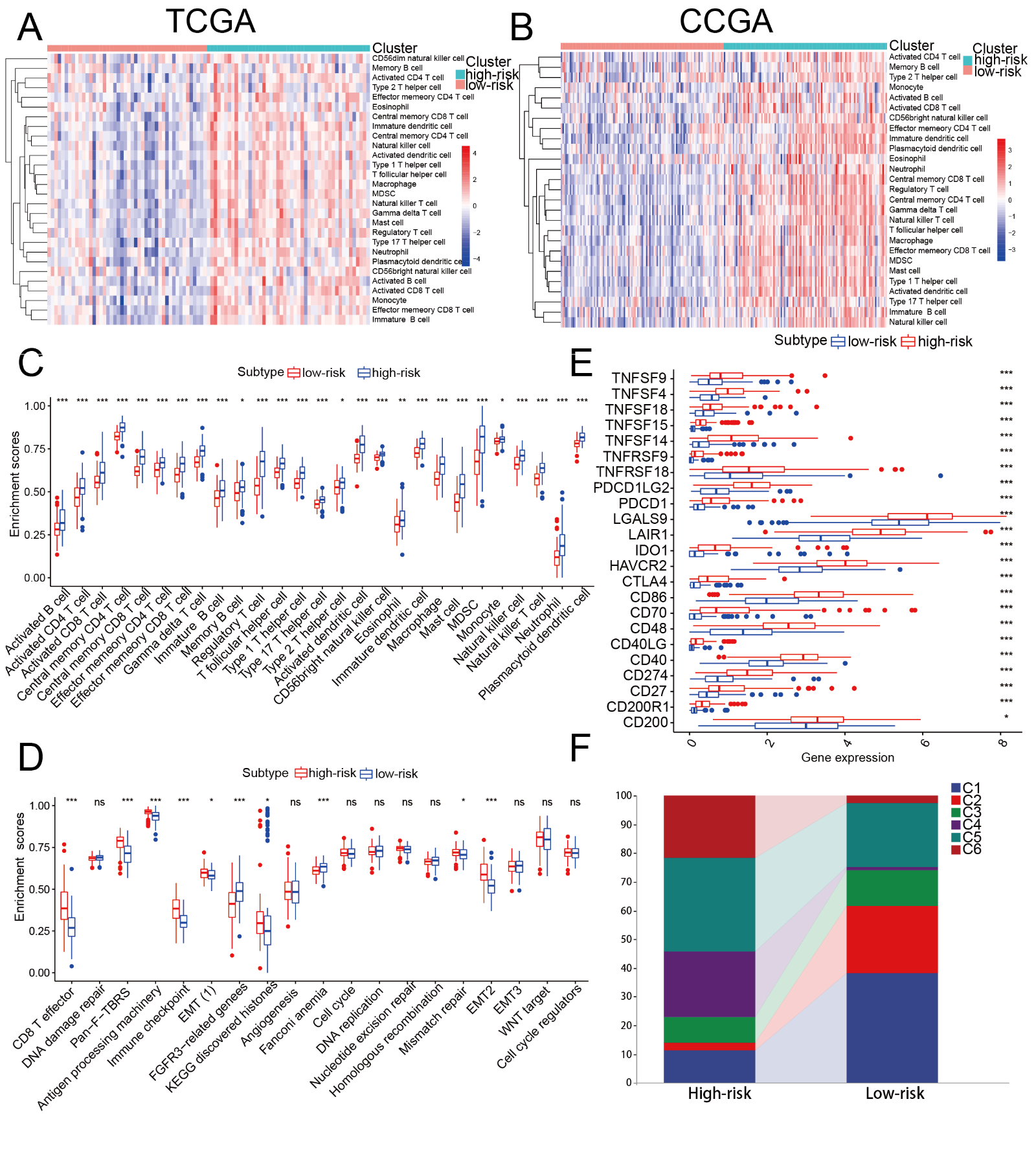


**Figure S6.** Immune infiltration analysis of IRPM in CCGA.

**(A,B)** The heatmap analysis of 22 immune cell divisions in TCGA and CGGA cohorts. **(C-E)** Quantitative analysis of the enrichment fraction of 22 immune cells, immune checkpoints, and immune-related pathways in two groups. **(F)** The proportion of six pan-cancer immune types in two groups in TCGA cohort.


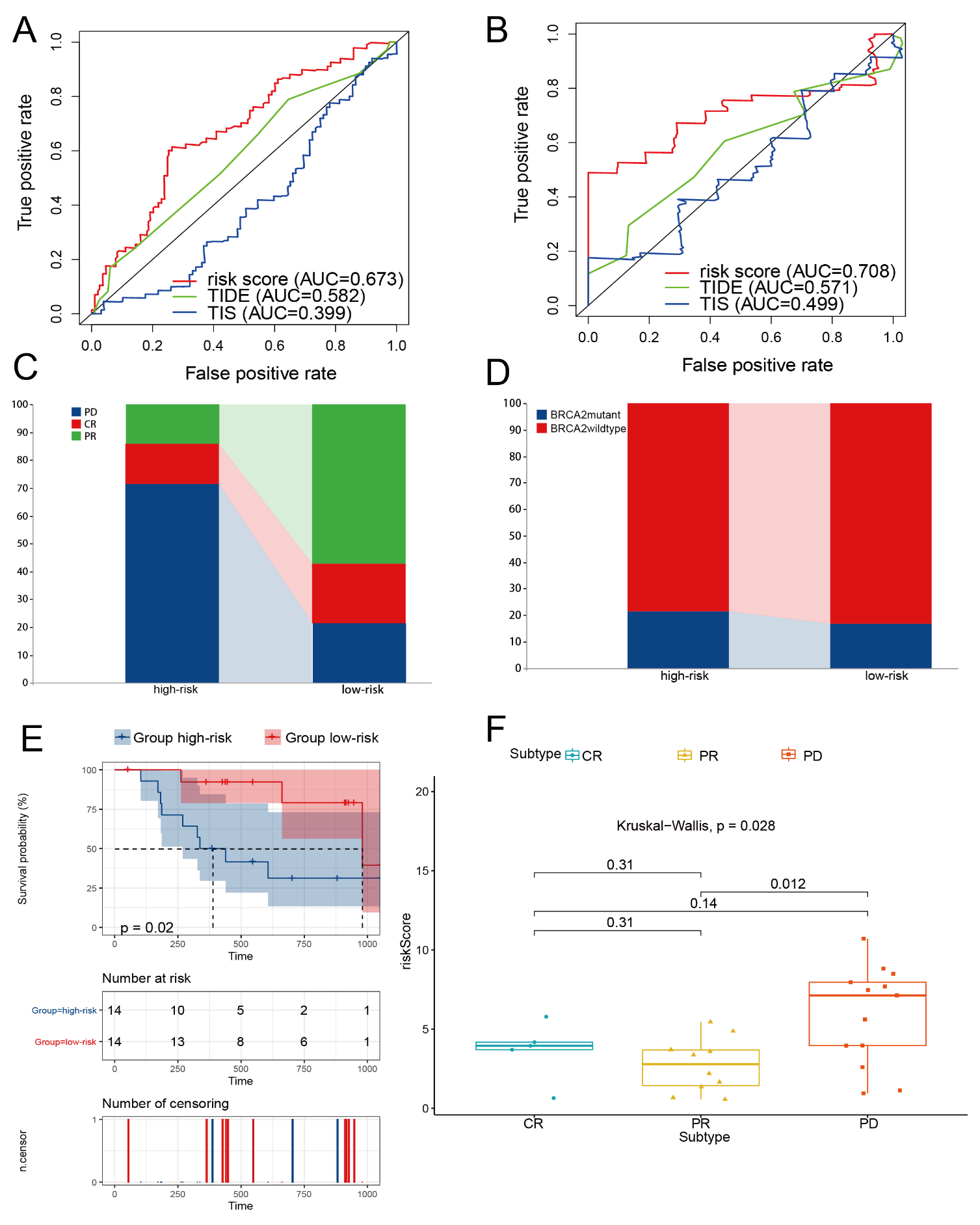


**Figure S7.** The correlation of high and low-risk groups with TIDE and TIS scores.

**(A,B)** ROC analysis of IRPM, TIS, and TIDE for overall survival at 1 year and 3 year follow-up in TCGA GBM cohort. **(C)** The percentage of patients in the GSE78220 cohort with clinical response in high-risk and low-risk groups. **(D)** Distinction in the risk between the *BRCA2* mutant and *BRCA2* wild type. **(E)** Survival analyses for the both groups using Kaplan–Meier analysis with the log-rank test. **(F)** Distinction in the risk among patients with distinct clinical responses in the GBM cohort. SD, stable disease; PD, progressive disease; CR, complete response; PR, partial response.


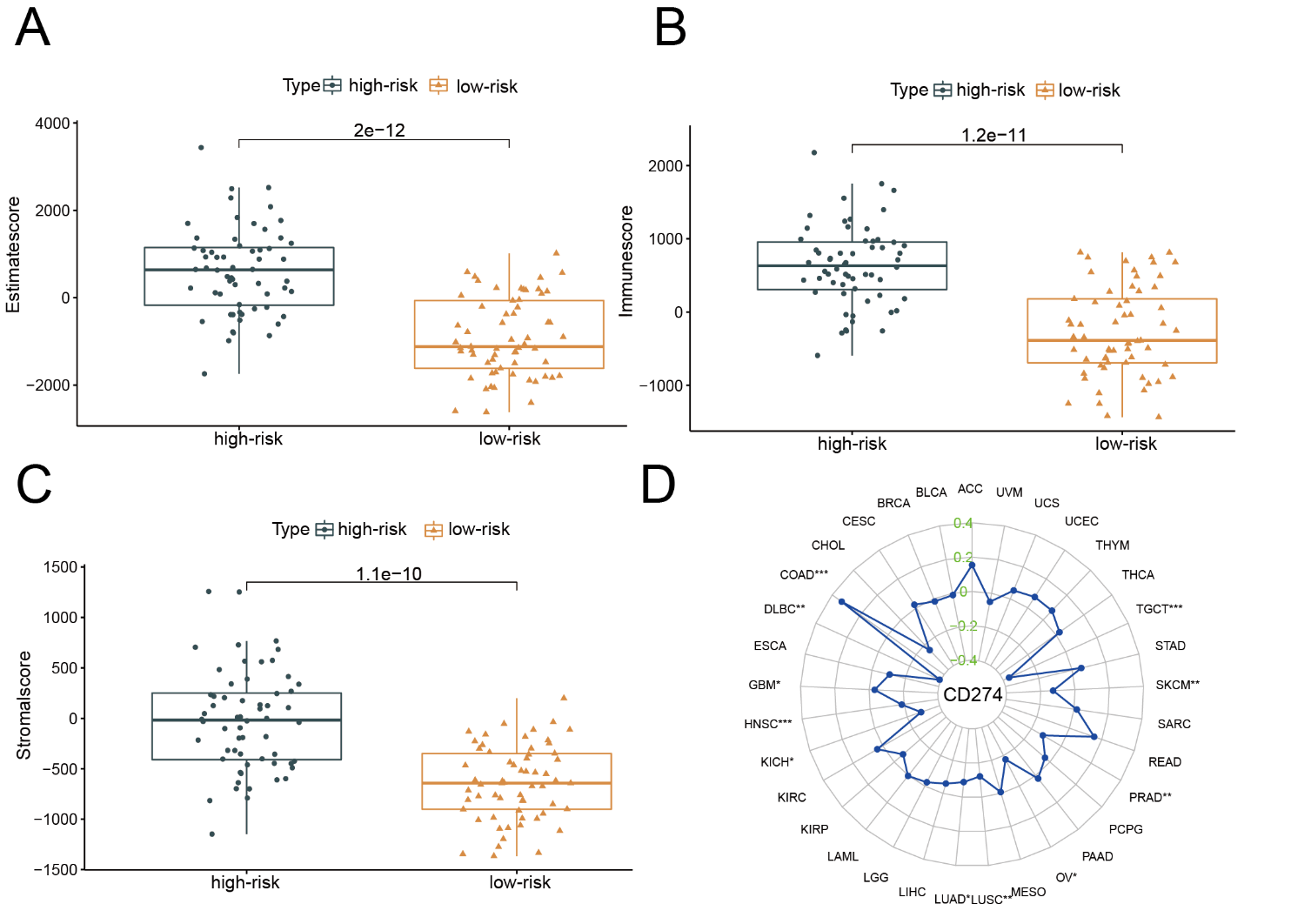


**Figure S8.** The correlation of high and low-risk groups with immunization scores.

**(A)** The relationship between both groups and Estimatescore. **(B)** The relationship between both groups and Immunescore. **(C)** The relationship between both groups and Stormalscore. **(D)** Correlation of PD-L1 and immune cells fraction for pan-cancer (Pearson test).


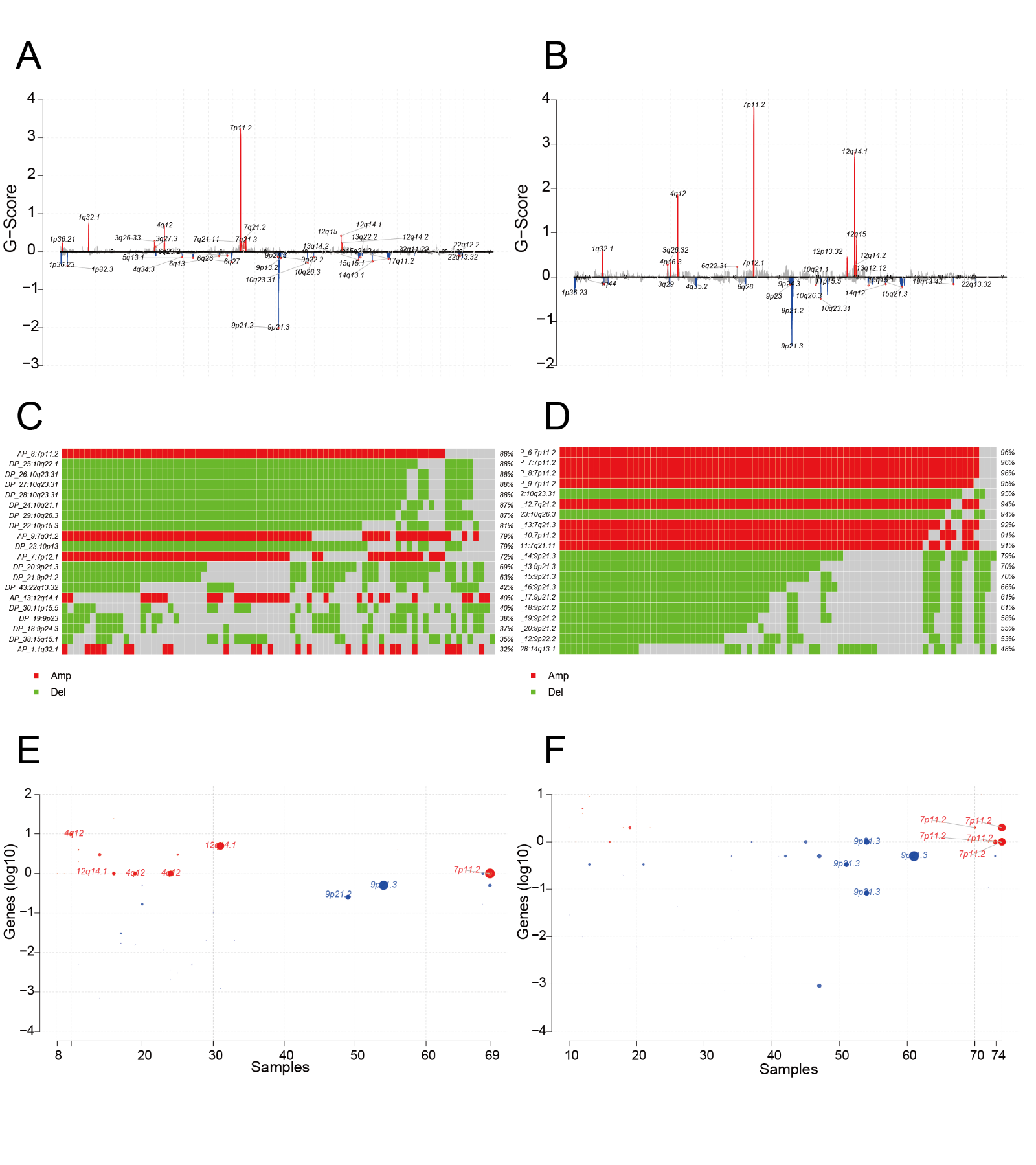


**Figure 9.** Comprehensive analyses of copy number variation between different risk groups.

**(A,B)** Significant amplifications and deletions of copy numbers were detected and compared between the two cohorts. **(C,D)** Copy number comparison of the top 20 loci of the two cohorts. **(E,F)** Distribution of Amp and Del in two cohort samples with high and low-risk.
