# Supplementary material for: Multiomics Data Analysis and Identification of Immune-Related Prognostic Signatures With Potential Implications in Prognosis and Immune Checkpoint Blockade Therapy of Glioblastoma
Source: Front Neurol. 2022 May 20;13:886913. doi: 10.3389/fneur.2022.886913 (PMC9165649; doi:10.3389/fneur.2022.886913)
Supplement: Supplementary file 2 [file Table_2.DOCX]

**Table S1.** Clinicopathological features of patients included in this study.

|  |  | TCGA dataset | | CGGA dataset | |
| --- | --- | --- | --- | --- | --- |
|  |  | Number | Percentage | Number | Percentage |
| Total |  | 174 | 100% | 388 | 100% |
| N/T |  | | | | |
|  | Normal | 5 | 2.87% | 0 |  |
|  | Tumor | 169 | 97.12% | 388 | 100% |
| Gender |  | | | | |
|  | Female | 59 | 34.91% | 153 | 39.43% |
|  | Male | 110 | 65.08% | 235 | 30.57% |
| Subtype |  | | | | |
|  | Classical | 42 | 25.45% | NA |  |
|  | Mesenchymal | 56 | 33.94% | NA |  |
|  | Neural | 28 | 16.97% | NA |  |
|  | Proneural | 39 | 23.64% | NA |  |
| P/R |  | | | | |
|  | Primary | 156 | 92.31% | 225 | 58.00% |
|  | Recurrence | 13 | 7.69% | 163 | 42.00% |
| Fustat |  | | | | |
|  | Dead | 118 | 69.82% | 289 | 81.875 |
|  | Alive | 51 | 30.18% | 64 | 18.13% |
| Age |  | | | | |
|  | <= 65 | 109 | 64.50 % | 29 | 7.47% |
|  | > 65 | 60 | 35.50 % | 359 | 92.53% |
| G-CIMP status |  |  |  |  |  |
|  | G-CIMP | 11 | 6.67% | NA |  |
|  | Non-G-CIMP | 154 | 93.32% | NA |  |
| IDH status |  | | | | |
|  | Mutant | 9 | 6.0% | 90 | 23.80% |
|  | Wildtype | 140 | 94.0% | 288 | 76.20% |
| 1p19q |  | | | | |
|  | Codel | NA | NA | 20 | 5.65% |
|  | Non-Codel | NA | NA | 334 | 94.35% |

Abbreviations: IDH, isocitrate dehydrogenase; G-CIMP, cytosine-phosphate-guanine island methylator phenotype; Codel, codeletion; NA, Not Applicable.

**Table S2.** GBM TIDE score.

| Patient | TIDE | Dysfunction | Exclusion | MSI Expr Sig |
| --- | --- | --- | --- | --- |
| TCGA-14-0871-01A | 0.27 | -0.1 | 0.27 | 0.95 |
| TCGA-28-5218-01A | 0.26 | -0.05 | 0.26 | 0.91 |
| TCGA-06-0644-01A | 0.25 | -0.06 | 0.25 | 0.92 |
| TCGA-06-2569-01A | 0.25 | -0.07 | 0.25 | 0.95 |
| TCGA-06-0190-02A | 0.25 | -0.06 | 0.25 | 0.92 |
| TCGA-02-0055-01A | 0.25 | -0.08 | 0.25 | 0.93 |
| TCGA-19-4065-02A | 0.24 | -0.06 | 0.24 | 0.94 |
| TCGA-26-5135-01A | 0.24 | -0.06 | 0.24 | 0.95 |
| TCGA-06-0138-01A | 0.24 | -0.11 | 0.24 | 0.94 |
| TCGA-06-0157-01A | 0.24 | -0.07 | 0.24 | 0.94 |
| TCGA-28-5209-01A | 0.24 | -0.07 | 0.24 | 0.94 |
| TCGA-32-4213-01A | 0.23 | -0.08 | 0.23 | 0.93 |
| TCGA-06-0745-01A | 0.23 | -0.1 | 0.23 | 0.94 |
| TCGA-06-0174-01A | 0.23 | -0.1 | 0.23 | 0.95 |
| TCGA-27-2524-01A | 0.23 | -0.08 | 0.23 | 0.93 |
| TCGA-76-4928-01B | 0.23 | -0.09 | 0.23 | 0.93 |
| TCGA-14-1825-01A | 0.23 | -0.11 | 0.23 | 0.96 |
| TCGA-19-2624-01A | 0.23 | -0.09 | 0.23 | 0.96 |
| TCGA-12-0616-01A | 0.22 | -0.09 | 0.22 | 0.94 |
| TCGA-06-5412-01A | 0.22 | -0.06 | 0.22 | 0.93 |
| TCGA-12-1597-01B | 0.22 | -0.1 | 0.22 | 0.95 |
| TCGA-28-5204-01A | 0.22 | -0.09 | 0.22 | 0.94 |
| TCGA-14-0781-01B | 0.22 | -0.08 | 0.22 | 0.92 |
| TCGA-32-1982-01A | 0.22 | -0.07 | 0.22 | 0.94 |
| TCGA-06-0190-01A | 0.22 | -0.07 | 0.22 | 0.94 |
| TCGA-06-2559-01A | 0.22 | -0.12 | 0.22 | 0.96 |
| TCGA-06-0744-01A | 0.22 | -0.09 | 0.22 | 0.95 |
| TCGA-27-2528-01A | 0.22 | -0.11 | 0.22 | 0.93 |
| TCGA-02-2483-01A | 0.22 | -0.08 | 0.22 | 0.94 |
| TCGA-26-5136-01B | 0.22 | -0.07 | 0.22 | 0.95 |
| TCGA-76-4927-01A | 0.21 | -0.07 | 0.21 | 0.94 |
| TCGA-06-0747-01A | 0.21 | -0.06 | 0.21 | 0.93 |
| TCGA-06-0686-01A | 0.21 | -0.09 | 0.21 | 0.96 |
| TCGA-27-2526-01A | 0.21 | -0.12 | 0.21 | 0.92 |
| TCGA-26-5133-01A | 0.21 | -0.11 | 0.21 | 0.94 |
| TCGA-06-5416-01A | 0.21 | -0.11 | 0.21 | 0.96 |
| TCGA-15-0742-01A | 0.21 | -0.08 | 0.21 | 0.94 |
| TCGA-28-1753-01A | 0.21 | -0.02 | 0.21 | 0.95 |
| TCGA-06-5413-01A | 0.21 | -0.07 | 0.21 | 0.95 |
| TCGA-41-3915-01A | 0.21 | -0.08 | 0.21 | 0.95 |
| TCGA-06-5418-01A | 0.21 | -0.1 | 0.21 | 0.94 |
| TCGA-14-1829-01A | 0.21 | -0.14 | 0.21 | 0.94 |
| TCGA-27-2521-01A | 0.21 | -0.12 | 0.21 | 0.95 |
| TCGA-12-3652-01A | 0.21 | -0.12 | 0.21 | 0.94 |
| TCGA-14-1034-01A | 0.21 | -0.07 | 0.21 | 0.93 |
| TCGA-12-3650-01A | 0.21 | -0.08 | 0.21 | 0.95 |
| TCGA-06-0750-01A | 0.21 | -0.06 | 0.21 | 0.94 |
| TCGA-76-4925-01A | 0.2 | -0.13 | 0.2 | 0.95 |
| TCGA-06-0130-01A | 0.2 | -0.04 | 0.2 | 0.93 |
| TCGA-27-1830-01A | 0.2 | -0.07 | 0.2 | 0.95 |
| TCGA-76-4931-01A | 0.2 | -0.08 | 0.2 | 0.96 |
| TCGA-02-0047-01A | 0.2 | -0.05 | 0.2 | 0.94 |
| TCGA-12-5299-01A | 0.2 | -0.09 | 0.2 | 0.94 |
| TCGA-12-0618-01A | 0.2 | -0.11 | 0.2 | 0.96 |
| TCGA-06-0156-01A | 0.2 | -0.07 | 0.2 | 0.95 |
| TCGA-32-2632-01A | 0.2 | -0.04 | 0.2 | 0.95 |
| TCGA-06-5856-01A | 0.2 | -0.1 | 0.2 | 0.94 |
| TCGA-76-4932-01A | 0.2 | -0.1 | 0.2 | 0.94 |
| TCGA-06-0238-01A | 0.2 | -0.11 | 0.2 | 0.95 |
| TCGA-28-2513-01A | 0.2 | -0.06 | 0.2 | 0.92 |
| TCGA-27-1832-01A | 0.2 | -0.05 | 0.2 | 0.95 |
| TCGA-28-5216-01A | 0.2 | -0.08 | 0.2 | 0.95 |
| TCGA-41-2572-01A | 0.2 | -0.07 | 0.2 | 0.94 |
| TCGA-06-0211-01B | 0.2 | -0.09 | 0.2 | 0.94 |
| TCGA-41-2571-01A | 0.2 | -0.1 | 0.2 | 0.95 |
| TCGA-12-0619-01A | 0.2 | -0.07 | 0.2 | 0.94 |
| TCGA-06-0187-01A | 0.2 | -0.07 | 0.2 | 0.93 |
| TCGA-06-0211-02A | 0.2 | -0.08 | 0.2 | 0.94 |
| TCGA-08-0386-01A | 0.2 | -0.1 | 0.2 | 0.95 |
| TCGA-06-2558-01A | 0.2 | -0.11 | 0.2 | 0.96 |
| TCGA-06-0878-01A | 0.2 | -0.09 | 0.2 | 0.94 |
| TCGA-06-0125-01A | 0.2 | -0.07 | 0.2 | 0.95 |
| TCGA-27-2523-01A | 0.2 | -0.1 | 0.2 | 0.94 |
| TCGA-06-0649-01B | 0.2 | -0.07 | 0.2 | 0.95 |
| TCGA-14-0790-01B | 0.2 | -0.09 | 0.2 | 0.95 |
| TCGA-19-2619-01A | 0.2 | -0.06 | 0.2 | 0.94 |
| TCGA-41-5651-01A | 0.2 | -0.11 | 0.2 | 0.96 |
| TCGA-19-2625-01A | 0.19 | -0.09 | 0.19 | 0.93 |
| TCGA-26-5139-01A | 0.19 | -0.07 | 0.19 | 0.94 |
| TCGA-14-1034-02B | 0.19 | -0.12 | 0.19 | 0.95 |
| TCGA-19-4065-01A | 0.19 | -0.1 | 0.19 | 0.95 |
| TCGA-28-2514-01A | 0.19 | -0.06 | 0.19 | 0.95 |
| TCGA-14-0817-01A | 0.19 | -0.08 | 0.19 | 0.94 |
| TCGA-28-5208-01A | 0.19 | -0.07 | 0.19 | 0.94 |
| TCGA-14-1402-02A | 0.19 | -0.12 | 0.19 | 0.95 |
| TCGA-06-0210-01A | 0.19 | -0.09 | 0.19 | 0.93 |
| TCGA-12-0821-01A | 0.19 | -0.1 | 0.19 | 0.94 |
| TCGA-06-0743-01A | 0.19 | -0.05 | 0.19 | 0.95 |
| TCGA-12-5295-01A | 0.19 | -0.11 | 0.19 | 0.93 |
| TCGA-06-5417-01A | 0.19 | -0.15 | 0.19 | 0.96 |
| TCGA-26-5134-01A | 0.19 | -0.09 | 0.19 | 0.96 |
| TCGA-06-0184-01A | 0.19 | -0.07 | 0.19 | 0.93 |
| TCGA-06-5408-01A | 0.19 | -0.08 | 0.19 | 0.95 |
| TCGA-28-2509-01A | 0.19 | -0.1 | 0.19 | 0.94 |
| TCGA-28-5215-01A | 0.19 | -0.06 | 0.19 | 0.95 |
| TCGA-06-0211-01A | 0.19 | -0.06 | 0.19 | 0.93 |
| TCGA-28-1747-01C | 0.19 | -0.06 | 0.19 | 0.94 |
| TCGA-27-1831-01A | 0.19 | -0.13 | 0.19 | 0.93 |
| TCGA-32-1970-01A | 0.19 | -0.08 | 0.19 | 0.95 |
| TCGA-32-2634-01A | 0.18 | -0.08 | 0.18 | 0.95 |
| TCGA-76-4926-01B | 0.18 | -0.07 | 0.18 | 0.94 |
| TCGA-02-2485-01A | 0.18 | -0.08 | 0.18 | 0.95 |
| TCGA-76-4929-01A | 0.18 | -0.08 | 0.18 | 0.95 |
| TCGA-06-5858-01A | 0.18 | -0.08 | 0.18 | 0.95 |
| TCGA-06-0210-02A | 0.18 | -0.05 | 0.18 | 0.93 |
| TCGA-14-0787-01A | 0.18 | -0.06 | 0.18 | 0.94 |
| TCGA-28-5213-01A | 0.18 | -0.09 | 0.18 | 0.94 |
| TCGA-27-1837-01A | 0.18 | -0.06 | 0.18 | 0.95 |
| TCGA-19-1389-02A | 0.18 | -0.08 | 0.18 | 0.91 |
| TCGA-06-2562-01A | 0.18 | -0.07 | 0.18 | 0.94 |
| TCGA-28-5220-01A | 0.18 | -0.09 | 0.18 | 0.95 |
| TCGA-06-2557-01A | 0.18 | -0.05 | 0.18 | 0.95 |
| TCGA-06-0141-01A | 0.18 | -0.09 | 0.18 | 0.93 |
| TCGA-19-2620-01A | 0.18 | -0.08 | 0.18 | 0.94 |
| TCGA-06-0125-02A | 0.18 | -0.06 | 0.18 | 0.93 |
| TCGA-06-0178-01A | 0.18 | -0.12 | 0.18 | 0.93 |
| TCGA-06-2567-01A | 0.18 | -0.06 | 0.18 | 0.95 |
| TCGA-26-5132-01A | 0.18 | -0.08 | 0.18 | 0.95 |
| TCGA-19-1390-01A | 0.17 | -0.08 | 0.17 | 0.96 |
| TCGA-32-2615-01A | 0.17 | -0.06 | 0.17 | 0.95 |
| TCGA-27-1835-01A | 0.17 | -0.11 | 0.17 | 0.93 |
| TCGA-06-0221-02A | 0.17 | -0.07 | 0.17 | 0.96 |
| TCGA-12-3653-01A | 0.17 | -0.08 | 0.17 | 0.93 |
| TCGA-14-2554-01A | 0.17 | -0.09 | 0.17 | 0.93 |
| TCGA-06-2564-01A | 0.17 | -0.08 | 0.17 | 0.95 |
| TCGA-27-2519-01A | 0.17 | -0.07 | 0.17 | 0.93 |
| TCGA-06-5411-01A | 0.17 | -0.08 | 0.17 | 0.96 |
| TCGA-41-4097-01A | 0.17 | -0.06 | 0.17 | 0.94 |
| TCGA-28-2510-01A | 0.17 | -0.07 | 0.17 | 0.95 |
| TCGA-32-2638-01A | 0.17 | -0.04 | 0.17 | 0.93 |
| TCGA-06-0219-01A | 0.17 | -0.13 | 0.17 | 0.93 |
| TCGA-06-5859-01A | 0.16 | -0.07 | 0.16 | 0.94 |
| TCGA-32-5222-01A | 0.16 | -0.1 | 0.16 | 0.94 |
| TCGA-06-5410-01A | 0.16 | -0.06 | 0.16 | 0.92 |
| TCGA-06-2570-01A | 0.16 | -0.07 | 0.16 | 0.96 |
| TCGA-14-0736-02A | 0.16 | -0.1 | 0.16 | 0.94 |
| TCGA-15-1444-01A | 0.16 | -0.07 | 0.16 | 0.95 |
| TCGA-06-0645-01A | 0.16 | -0.05 | 0.16 | 0.92 |
| TCGA-06-0168-01A | 0.16 | -0.06 | 0.16 | 0.94 |
| TCGA-06-0646-01A | 0.16 | -0.05 | 0.16 | 0.95 |
| TCGA-06-2561-01A | 0.16 | -0.08 | 0.16 | 0.94 |
| TCGA-14-1823-01A | 0.16 | -0.11 | 0.16 | 0.92 |
| TCGA-28-5207-01A | 0.16 | -0.07 | 0.16 | 0.95 |
| TCGA-06-0882-01A | 0.16 | -0.06 | 0.16 | 0.93 |
| TCGA-06-2563-01A | 0.16 | -0.07 | 0.16 | 0.93 |
| TCGA-27-1834-01A | 0.16 | -0.06 | 0.16 | 0.95 |
| TCGA-19-5960-01A | 0.16 | -0.1 | 0.16 | 0.95 |
| TCGA-19-1787-01B | 0.16 | -0.07 | 0.16 | 0.94 |
| TCGA-06-5414-01A | 0.15 | -0.07 | 0.15 | 0.93 |
| TCGA-26-1442-01A | 0.15 | -0.09 | 0.15 | 0.95 |
| TCGA-16-1045-01B | 0.15 | -0.06 | 0.15 | 0.93 |
| TCGA-06-0139-01A | 0.15 | -0.07 | 0.15 | 0.92 |
| TCGA-06-2565-01A | 0.15 | -0.05 | 0.15 | 0.94 |
| TCGA-06-0132-01A | 0.15 | -0.06 | 0.15 | 0.93 |
| TCGA-06-0158-01A | 0.15 | -0.04 | 0.15 | 0.94 |
| TCGA-28-2499-01A | 0.15 | -0.09 | 0.15 | 0.93 |
| TCGA-14-0789-01A | 0.15 | -0.07 | 0.15 | 0.92 |
| TCGA-06-0152-02A | 0.15 | -0.03 | 0.15 | 0.94 |
| TCGA-16-0846-01A | 0.15 | -0.05 | 0.15 | 0.94 |
| TCGA-32-1980-01A | 0.15 | -0.06 | 0.15 | 0.94 |
| TCGA-19-2629-01A | 0.15 | -0.08 | 0.15 | 0.94 |
| TCGA-06-1804-01A | 0.15 | -0.06 | 0.15 | 0.97 |
| TCGA-19-0957-02A | 0.15 | -0.08 | 0.15 | 0.94 |
| TCGA-32-2616-01A | 0.14 | -0.08 | 0.14 | 0.95 |
| TCGA-02-2486-01A | 0.14 | -0.06 | 0.14 | 0.92 |
| TCGA-06-0749-01A | 0.14 | -0.06 | 0.14 | 0.94 |
| TCGA-06-0129-01A | 0.11 | -0.09 | 0.11 | 0.96 |
| TCGA-06-0681-11A | 0.11 | -0.06 | 0.11 | 0.95 |
| TCGA-06-0680-11A | 0.1 | -0.06 | 0.1 | 0.94 |
| TCGA-06-AABW-11A | 0.1 | -0.09 | 0.1 | 0.93 |
| TCGA-06-0675-11A | 0.1 | -0.06 | 0.1 | 0.94 |
| TCGA-06-0171-02A | 0.09 | -0.02 | 0.09 | 0.92 |
| TCGA-06-0678-11A | 0.09 | -0.06 | 0.09 | 0.94 |

**Table S3.** GBM Estimate score.

| ID | Platform | Stromal score | Immune score | ESTIMATE score | Tumor.purity |
| --- | --- | --- | --- | --- | --- |
| TCGA.02.0047.01 | glioblastoma multiforme | 133.88 | 513.41 | 647.3 | 0.68 |
| TCGA.02.2483.01 | glioblastoma multiforme | -465.15 | 86.9 | -378.25 | 0.72 |
| TCGA.02.2485.01 | glioblastoma multiforme | -714.18 | -515.07 | -1229.26 | 0.93 |
| TCGA.02.2486.01 | glioblastoma multiforme | 105.42 | 1661.3 | 1766.72 | 0.72 |
| TCGA.06.0125.01 | glioblastoma multiforme | -657.33 | -647.46 | -1304.78 | 0.89 |
| TCGA.06.0129.01 | glioblastoma multiforme | -760.95 | 695.79 | -65.17 | 0.85 |
| TCGA.06.0130.01 | glioblastoma multiforme | 1257.82 | 2176.81 | 3434.63 | 0.27 |
| TCGA.06.0138.01 | glioblastoma multiforme | 234.34 | 846.09 | 1080.44 | 0.71 |
| TCGA.06.0157.01 | glioblastoma multiforme | -39.83 | 182.27 | 142.44 | 0.83 |
| TCGA.06.0158.01 | glioblastoma multiforme | -356.28 | 443.07 | 86.79 | 0.79 |
| TCGA.06.0168.01 | glioblastoma multiforme | 338.12 | 906.28 | 1244.4 | 0.68 |
| TCGA.06.0174.01 | glioblastoma multiforme | -791.82 | -622.36 | -1414.17 | 0.95 |
| TCGA.06.0187.01 | glioblastoma multiforme | -318.1 | 402.61 | 84.51 | 0.87 |
| TCGA.06.0190.01 | glioblastoma multiforme | 704.72 | 994.95 | 1699.67 | 0.58 |
| TCGA.06.0211.01 | glioblastoma multiforme | -697.64 | -285.73 | -983.37 | 0.87 |
| TCGA.06.0649.01 | glioblastoma multiforme | 414.24 | 952.91 | 1367.15 | 0.63 |
| TCGA.06.0686.01 | glioblastoma multiforme | -613.71 | -170.55 | -784.26 | 0.74 |
| TCGA.06.0743.01 | glioblastoma multiforme | -448.48 | 17.61 | -430.87 | 0.85 |
| TCGA.06.0744.01 | glioblastoma multiforme | -809.05 | -339.16 | -1148.21 | 0.9 |
| TCGA.06.0745.01 | glioblastoma multiforme | -216.14 | 436.08 | 219.94 | 0.82 |
| TCGA.06.0747.01 | glioblastoma multiforme | -1148 | -592.5 | -1740.5 | 0.92 |
| TCGA.06.0749.01 | glioblastoma multiforme | -490.36 | 713.51 | 223.15 | 0.56 |
| TCGA.06.0750.01 | glioblastoma multiforme | 268.12 | 613.66 | 881.78 | 0.72 |
| TCGA.06.0878.01 | glioblastoma multiforme | 14.12 | 675.4 | 689.52 | 0.76 |
| TCGA.06.0882.01 | glioblastoma multiforme | -0.32 | 882.89 | 882.57 | 0.52 |
| TCGA.06.1804.01 | glioblastoma multiforme | -537.32 | -387.33 | -924.65 | 0.82 |
| TCGA.06.2557.01 | glioblastoma multiforme | 46.33 | 1318.65 | 1364.97 | 0.72 |
| TCGA.06.2558.01 | glioblastoma multiforme | -182.59 | -36.54 | -219.13 | 0.82 |
| TCGA.06.2559.01 | glioblastoma multiforme | -435.42 | -138.92 | -574.34 | 0.9 |
| TCGA.06.2561.01 | glioblastoma multiforme | -227.29 | 814.99 | 587.7 | 0.63 |
| TCGA.06.2562.01 | glioblastoma multiforme | 9.16 | 375.12 | 384.28 | 0.74 |
| TCGA.06.2563.01 | glioblastoma multiforme | -467.09 | -168.49 | -635.58 | 0.86 |
| TCGA.06.2564.01 | glioblastoma multiforme | -247.15 | 339.71 | 92.55 | 0.78 |
| TCGA.06.2565.01 | glioblastoma multiforme | -643.4 | -254.04 | -897.44 | 0.91 |
| TCGA.06.2567.01 | glioblastoma multiforme | -112.17 | 680.43 | 568.26 | 0.8 |
| TCGA.06.2569.01 | glioblastoma multiforme | -36.78 | -1434.95 | -1471.73 | 0.93 |
| TCGA.06.2570.01 | glioblastoma multiforme | -298.92 | 487.01 | 188.08 | 0.89 |
| TCGA.06.5408.01 | glioblastoma multiforme | -545.84 | -258.71 | -804.54 | 0.91 |
| TCGA.06.5411.01 | glioblastoma multiforme | -353.77 | -34.56 | -388.33 | 0.63 |
| TCGA.06.5412.01 | glioblastoma multiforme | 728.17 | 1554.21 | 2282.38 | 0.28 |
| TCGA.06.5413.01 | glioblastoma multiforme | 341.24 | 588.11 | 929.34 | 0.74 |
| TCGA.06.5414.01 | glioblastoma multiforme | -318.85 | 802.85 | 484 | 0.78 |
| TCGA.06.5415.01 | glioblastoma multiforme | -747.38 | -604.19 | -1351.57 | 0.83 |
| TCGA.06.5416.01 | glioblastoma multiforme | -1333.69 | -1069.91 | -2403.59 | 0.78 |
| TCGA.06.5418.01 | glioblastoma multiforme | -404.08 | 518.25 | 114.17 | 0.8 |
| TCGA.06.5858.01 | glioblastoma multiforme | -113.13 | 285.19 | 172.07 | 0.85 |
| TCGA.06.5859.01 | glioblastoma multiforme | -357.41 | 570.43 | 213.02 | 0.79 |
| TCGA.12.0821.01 | glioblastoma multiforme | -622.46 | -689.08 | -1311.53 | 0.9 |
| TCGA.12.3650.01 | glioblastoma multiforme | -902.15 | -114.29 | -1016.44 | 0.88 |
| TCGA.12.3652.01 | glioblastoma multiforme | -1196.44 | -509.25 | -1705.69 | 0.92 |
| TCGA.12.3653.01 | glioblastoma multiforme | -815.92 | 270.77 | -545.15 | 0.92 |
| TCGA.12.5295.01 | glioblastoma multiforme | -424.66 | 802.55 | 377.9 | 0.73 |
| TCGA.12.5299.01 | glioblastoma multiforme | -446.51 | 228.37 | -218.13 | 0.92 |
| TCGA.14.0787.01 | glioblastoma multiforme | -699.19 | 318.72 | -380.47 | 0.84 |
| TCGA.14.0789.01 | glioblastoma multiforme | 174.9 | 1163.11 | 1338.01 | 0.56 |
| TCGA.14.0790.01 | glioblastoma multiforme | -944.8 | -980.16 | -1924.96 | 0.93 |
| TCGA.14.0871.01 | glioblastoma multiforme | -643.33 | -1418.34 | -2061.67 | 0.92 |
| TCGA.14.1034.01 | glioblastoma multiforme | -101.6 | 732.79 | 631.19 | 0.55 |
| TCGA.14.1823.01 | glioblastoma multiforme | -10.08 | 1145.51 | 1135.43 | 0.62 |
| TCGA.14.1825.01 | glioblastoma multiforme | -1061.36 | -692 | -1753.36 | 0.89 |
| TCGA.14.1829.01 | glioblastoma multiforme | 199.11 | 812.78 | 1011.89 | 0.71 |
| TCGA.14.2554.01 | glioblastoma multiforme | -195.99 | 649.56 | 453.57 | 0.81 |
| TCGA.15.0742.01 | glioblastoma multiforme | -1094.15 | -633.68 | -1727.84 | 0.93 |
| TCGA.15.1444.01 | glioblastoma multiforme | -159.96 | 545.12 | 385.16 | 0.61 |
| TCGA.16.1045.01 | glioblastoma multiforme | 683.48 | 1397.17 | 2080.65 | 0.66 |
| TCGA.19.1390.01 | glioblastoma multiforme | -1274.25 | -762.16 | -2036.4 | 0.9 |
| TCGA.19.1787.01 | glioblastoma multiforme | -396.33 | 348.5 | -47.83 | 0.86 |
| TCGA.19.2619.01 | glioblastoma multiforme | -528.01 | -617.69 | -1145.7 | 0.88 |
| TCGA.19.2620.01 | glioblastoma multiforme | -409.41 | -157.98 | -567.39 | 0.67 |
| TCGA.19.2624.01 | glioblastoma multiforme | -1365.77 | -1251.46 | -2617.23 | 0.94 |
| TCGA.19.2625.01 | glioblastoma multiforme | -610.21 | -257.19 | -867.4 | 0.65 |
| TCGA.19.2629.01 | glioblastoma multiforme | -348.72 | 492.77 | 144.06 | 0.62 |
| TCGA.19.5960.01 | glioblastoma multiforme | -1345.85 | -1249.9 | -2595.74 | 0.89 |
| TCGA.26.1442.01 | glioblastoma multiforme | -816.46 | -431.69 | -1248.15 | 0.79 |
| TCGA.26.5132.01 | glioblastoma multiforme | -460.49 | -53.53 | -514.02 | 0.89 |
| TCGA.26.5133.01 | glioblastoma multiforme | -900.19 | -886.47 | -1786.66 | 0.89 |
| TCGA.26.5134.01 | glioblastoma multiforme | -773.37 | -345.83 | -1119.2 | 0.92 |
| TCGA.26.5135.01 | glioblastoma multiforme | -54.4 | 278.39 | 223.99 | 0.81 |
| TCGA.26.5136.01 | glioblastoma multiforme | -31.13 | 676.64 | 645.51 | 0.83 |
| TCGA.26.5139.01 | glioblastoma multiforme | -336.22 | 134.03 | -202.18 | 0.8 |
| TCGA.27.1830.01 | glioblastoma multiforme | 217.81 | 460.51 | 678.33 | 0.68 |
| TCGA.27.1831.01 | glioblastoma multiforme | 125.86 | 805.88 | 931.75 | 0.7 |
| TCGA.27.1832.01 | glioblastoma multiforme | 125.03 | 967.85 | 1092.88 | 0.6 |
| TCGA.27.1834.01 | glioblastoma multiforme | -217.49 | 677.94 | 460.44 | 0.75 |
| TCGA.27.1835.01 | glioblastoma multiforme | -851.64 | -954.37 | -1806 | 0.87 |
| TCGA.27.1837.01 | glioblastoma multiforme | -519.74 | 150.58 | -369.17 | 0.82 |
| TCGA.27.2519.01 | glioblastoma multiforme | 94.58 | 969.77 | 1064.35 | 0.68 |
| TCGA.27.2521.01 | glioblastoma multiforme | -994.88 | -897.69 | -1892.57 | 0.93 |
| TCGA.27.2523.01 | glioblastoma multiforme | -1087.81 | -527.19 | -1615 | 0.92 |
| TCGA.27.2524.01 | glioblastoma multiforme | -190.62 | 489.66 | 299.04 | 0.81 |
| TCGA.27.2526.01 | glioblastoma multiforme | -790.3 | 455.47 | -334.83 | 0.85 |
| TCGA.27.2528.01 | glioblastoma multiforme | -299.82 | -907.88 | -1207.7 | 0.76 |
| TCGA.28.1747.01 | glioblastoma multiforme | -550.81 | 746.75 | 195.94 | 0.8 |
| TCGA.28.1753.01 | glioblastoma multiforme | 574.4 | 989.75 | 1564.15 | 0.62 |
| TCGA.28.2510.01 | glioblastoma multiforme | -220.36 | -546.76 | -767.13 | 0.28 |
| TCGA.28.2513.01 | glioblastoma multiforme | 566.7 | 1267.34 | 1834.04 | 0.32 |
| TCGA.28.2514.01 | glioblastoma multiforme | -672.55 | -491.1 | -1163.65 | 0.88 |
| TCGA.28.5204.01 | glioblastoma multiforme | -598.67 | -4.25 | -602.92 | 0.89 |
| TCGA.28.5207.01 | glioblastoma multiforme | -25.38 | -131.84 | -157.22 | 0.76 |
| TCGA.28.5208.01 | glioblastoma multiforme | -130.81 | 179.63 | 48.82 | 0.86 |
| TCGA.28.5209.01 | glioblastoma multiforme | -639 | -528.09 | -1167.09 | 0.94 |
| TCGA.28.5213.01 | glioblastoma multiforme | 767.22 | 1751.71 | 2518.92 | 0.49 |
| TCGA.28.5218.01 | glioblastoma multiforme | 1252.76 | 1240.2 | 2492.96 | 0.6 |
| TCGA.28.5220.01 | glioblastoma multiforme | -638.29 | 252.38 | -385.9 | 0.88 |
| TCGA.32.1970.01 | glioblastoma multiforme | -672.06 | -395.76 | -1067.81 | 0.87 |
| TCGA.32.1982.01 | glioblastoma multiforme | 245.73 | 879.07 | 1124.8 | 0.58 |
| TCGA.32.2615.01 | glioblastoma multiforme | -94.15 | 516.03 | 421.87 | 0.84 |
| TCGA.32.2616.01 | glioblastoma multiforme | -618.79 | 55.7 | -563.1 | 0.82 |
| TCGA.32.2634.01 | glioblastoma multiforme | -762.61 | -729.05 | -1491.66 | 0.89 |
| TCGA.32.2638.01 | glioblastoma multiforme | 204.53 | 719.4 | 923.93 | 0.82 |
| TCGA.32.4213.01 | glioblastoma multiforme | 560.91 | 1135.71 | 1696.62 | 0.69 |
| TCGA.32.5222.01 | glioblastoma multiforme | -264.21 | 744.71 | 480.5 | 0.72 |
| TCGA.41.2571.01 | glioblastoma multiforme | -992.4 | -505.5 | -1497.9 | 0.78 |
| TCGA.41.2572.01 | glioblastoma multiforme | -402.08 | 148.55 | -253.52 | 0.84 |
| TCGA.41.3915.01 | glioblastoma multiforme | 389.01 | 797.81 | 1186.83 | 0.77 |
| TCGA.41.4097.01 | glioblastoma multiforme | 482.97 | 555.63 | 1038.61 | 0.52 |
| TCGA.41.5651.01 | glioblastoma multiforme | -984.52 | -1105.5 | -2090.01 | 0.94 |
| TCGA.76.4925.01 | glioblastoma multiforme | -1008.89 | -839.98 | -1848.86 | 0.94 |
| TCGA.76.4926.01 | glioblastoma multiforme | -818.01 | -424.35 | -1242.37 | 0.79 |
| TCGA.76.4928.01 | glioblastoma multiforme | -181.66 | 507.12 | 325.47 | 0.57 |
| TCGA.76.4929.01 | glioblastoma multiforme | -536.87 | -244.23 | -781.1 | 0.8 |
| TCGA.76.4931.01 | glioblastoma multiforme | -1115.19 | -717.12 | -1832.31 | 0.93 |
| TCGA.76.4932.01 | glioblastoma multiforme | -914.08 | -44.37 | -958.45 | 0.92 |

**Table S4.** Univariate regression results.

|  | gene | HR | z | 95%CI | pvalue |
| --- | --- | --- | --- | --- | --- |
| 1 | ADIPOQ | 1.11 | 0.44 | 0.69-1.8 | 0.663114 |
| 2 | CASP4 | 1.44 | 1.93 | 0.99-2.07 | 0.053321 |
| 3 | CFTR | 1.15 | 1.11 | 0.9-1.47 | 0.265772 |
| 4 | FGF7P3 | 1.03 | 0.16 | 0.75-1.4 | 0.871464 |
| 5 | GDF6 | 1.07 | 0.57 | 0.85-1.36 | 0.565812 |
| 6 | HMOX1 | 1.19 | 1.21 | 0.9-1.58 | 0.226264 |
| 7 | IL1R1 | 1.54 | 1.96 | 1-2.38 | 0.049752 |
| 8 | JAM3 | 1.24 | 0.79 | 0.73-2.12 | 0.429739 |
| 9 | LY86 | 1.05 | 0.41 | 0.83-1.32 | 0.683557 |
| 10 | MAP3K7 | 1.38 | 1.2 | 0.81-2.34 | 0.231085 |
| 11 | NLRP6 | 0.99 | -0.04 | 0.77-1.28 | 0.965605 |
| 12 | OAS1 | 1.32 | 1.84 | 0.98-1.76 | 0.066067 |
| 13 | PDGFB | 0.96 | -0.15 | 0.53-1.72 | 0.882895 |
| 14 | PROCR | 1.18 | 1.05 | 0.86-1.62 | 0.293412 |
| 15 | PSTPIP1 | 1.27 | 0.92 | 0.76-2.1 | 0.358525 |
| 16 | RAC2 | 1.22 | 1.28 | 0.9-1.66 | 0.20166 |
| 17 | RNF135 | 1.54 | 1.84 | 0.97-2.43 | 0.065328 |
| 18 | SCN5A | 1.03 | 0.23 | 0.78-1.36 | 0.821066 |
| 19 | SCT | 1.21 | 1.04 | 0.85-1.71 | 0.296106 |
| 20 | SPI1 | 1.22 | 1.33 | 0.91-1.62 | 0.185055 |
| 21 | TGFA | 0.86 | -0.98 | 0.63-1.17 | 0.326419 |
| 22 | TLR6 | 0.92 | -0.31 | 0.53-1.6 | 0.757809 |
| 23 | TNFAIP3 | 1.01 | 0.03 | 0.68-1.48 | 0.973335 |
| 24 | TNFSF12 | 1.64 | 2.09 | 1.03-2.61 | 0.036504 |
| 25 | TREM1 | 1.18 | 1.7 | 0.97-1.43 | 0.089158 |
| 26 | TYMP | 1.1 | 0.74 | 0.86-1.41 | 0.457658 |
| 27 | VDR | 1.51 | 2.02 | 1.01-2.24 | 0.043059 |
| 28 | WAS | 1.11 | 0.69 | 0.82-1.51 | 0.488642 |

**Table S5.** Correlation of Response ups with riskscore (GSE78220).

| id | riskscore | Subtype |
| --- | --- | --- |
| Pt13 | 15.41201425 | Complete Response |
| Pt27A | 18.00475954 | Complete Response |
| Pt27B | 1.562025605 | Complete Response |
| Pt8 | 12.98412832 | Complete Response |
| Pt9 | 54.95703221 | Complete Response |
| Pt15 | 2.556445475 | Partial Response |
| Pt19 | 10.33262504 | Partial Response |
| Pt2 | 11.98779618 | Partial Response |
| Pt28 | 43.789 | Partial Response |
| Pt35 | 1.594642885 | Partial Response |
| Pt37 | 3.153007559 | Partial Response |
| Pt38 | 13.02134217 | Partial Response |
| Pt4 | 1.476020278 | Partial Response |
| Pt5 | 4.572292496 | Partial Response |
| Pt6 | 29.24108876 | Partial Response |
| Pt1 | 249.0378231 | Progressive Disease |
| Pt10 | 359.4457998 | Progressive Disease |
| Pt12 | 176.9942284 | Progressive Disease |
| Pt14 | 6.034901492 | Progressive Disease |
| Pt16 | 452.7438041 | Progressive Disease |
| Pt20 | 48.7768142 | Progressive Disease |
| Pt22 | 206.4682171 | Progressive Disease |
| Pt23 | 15.47512168 | Progressive Disease |
| Pt25 | 2.181998801 | Progressive Disease |
| Pt29 | 15.55654638 | Progressive Disease |
| Pt31 | 139.5516765 | Progressive Disease |
| Pt32 | 1678.164786 | Progressive Disease |
| Pt7 | 1.921798595 | Progressive Disease |
